# CCR2^+^ Inflammatory Dendritic Cells and Translocation of Antigen by Type III Secretion Are Required for the Exceptionally Large CD8^+^ T Cell Response to the Protective YopE_69-77_ Epitope during *Yersinia* Infection

**DOI:** 10.1371/journal.ppat.1005167

**Published:** 2015-10-15

**Authors:** Yue Zhang, Jason W. Tam, Patricio Mena, Adrianus W. M. van der Velden, James B. Bliska

**Affiliations:** Center for Infectious Diseases and Department of Molecular Genetics and Microbiology, Stony Brook University, Stony Brook, New York, United States of America; University of California, Davis, UNITED STATES

## Abstract

During *Yersinia pseudotuberculosis* infection of C57BL/6 mice, an exceptionally large CD8^+^ T cell response to a protective epitope in the type III secretion system effector YopE is produced. At the peak of the response, up to 50% of splenic CD8^+^ T cells recognize the epitope YopE_69-77_. The features of the interaction between pathogen and host that result in this large CD8^+^ T cell response are unknown. Here, we used *Y*. *pseudotuberculosis* strains defective for production, secretion and/or translocation of YopE to infect wild-type or mutant mice deficient in specific dendritic cells (DCs). Bacterial colonization of organs and translocation of YopE into spleen cells was measured, and flow cytometry and tetramer staining were used to characterize the cellular immune response. We show that the splenic YopE_69-77_-specific CD8^+^ T cells generated during the large response are polyclonal and are produced by a “translocation-dependent” pathway that requires injection of YopE into host cell cytosol. Additionally, a smaller YopE_69-77_-specific CD8^+^ T cell response (~10% of the large expansion) can be generated in a “translocation-independent” pathway in which CD8α^+^ DCs cross present secreted YopE. CCR2-expressing inflammatory DCs were required for the large YopE_69-77_-specific CD8^+^ T cell expansion because this response was significantly reduced in Ccr2^-/-^ mice, YopE was translocated into inflammatory DCs in vivo, inflammatory DCs purified from infected spleens activated YopE_69-77_-specific CD8^+^ T cells ex vivo and promoted the expansion of YopE_69-77_-specific CD8^+^ T cells in infected Ccr2^-/-^ mice after adoptive transfer. A requirement for inflammatory DCs in producing a protective CD8^+^ T cell response to a bacterial antigen has not previously been demonstrated. Therefore, the production of YopE_69-77_-specific CD8^+^ T cells by inflammatory DCs that are injected with YopE during *Y*. *pseudotuberculosis* infection represents a novel mechanism for generating a massive and protective adaptive immune response.

## Author Summary

Dendritic cells (DCs) direct host protective adaptive immune responses during infection. How different subpopulations of DCs contribute to the formation of antigen-specific CD8^+^ T cells is incompletely understood. Infection of C57BL/6 mice with the bacterial pathogen *Yersinia pseudotuberculosis* results in the production of an exceptionally large CD8^+^ T cell response to an epitope in the type III secretion system effector YopE. Here, we show that this large CD8^+^ T cell response requires translocation of YopE into inflammatory DCs, which express CCR2 and accumulate in infected tissues. In contrast, when mice are infected with a *Y*. *pseudotuberculosis* strain that can secrete but not translocate YopE, a smaller response is seen, and under these conditions the generation of YopE-specific CD8^+^ T cell requires CD8α^+^ DCs. Our results indicate that distinct DC subsets participate in constructing the CD8^+^ T cell response to secreted, versus translocated, YopE. Furthermore our data indicate that inflammatory DCs are a driving force behind the massive CD8^+^ T cell response to a protective epitope in a bacterial virulence factor that is translocated into host cells.

## Introduction

Dendritic cells (DCs) play a major role in protective immunity against pathogens. For example, DCs are required to prime naïve antigen specific CD8^+^ T cells to become effector cells that secrete cytokines and/or are cytolytic [1,2]. When DCs acquire endogenous antigens, e.g., viral polypeptides synthesized intracellularly, the antigens are processed through a classical pathway. In this case, antigenic proteins are first degraded by the proteasome, then the peptide products are transported from cytosol through the endoplasmic reticulum to load onto MHC class I molecules and finally transported to the cell surface for presentation to CD8^+^ T cells [3]. In addition, when DCs are not directly infected, they can acquire exogenous antigens, e.g. from extracellular infectious agents, or antigens associated with other types of cells, and present them to CD8^+^ T cells by a mechanism known as cross-presentation. The two main intracellular pathways for cross-presentation are generally referred to as the cytosolic pathway, where the antigen is internalized and gains access to the cytosol, and the vacuolar pathway, where antigen processing and loading occurs in endocytic compartments [4].

DCs are a heterogeneous population of professional antigen presenting cells. They differ in hematological origin, migration pathway, surface marker expression and functional properties [5]. Originally DCs were identified to bear the surface marker CD11c [6]. Currently, common features of all DCs are still somewhat obscure but in general include a probing dendritic morphology, high amount of surface MHC class II molecules and T cell-stimulating activity [7]. At steady state, plasmacytoid DCs and conventional DCs are the main types. In mice, conventional DCs include lymphoid organ-resident and migratory subpopulations. The resident murine DCs can be further divided into CD8α^+^CD11b^low^ and CD8α^-^CD11b^+^ cells, while the migratory DCs can be separated into CD103^+^CD11b^low^ and CD103^-^CD11b^+^ cells. The CD8α^+^ and the CD103^+^ DCs are more efficient at cross-presentation in vivo and they are developmentally related. Deficiency in transcription factor Batf3 in mice results in the diminishment of both subpopulations of DCs [8,9].

During infection or tissue injury, another type of DC, inflammatory DC (infDC), may emerge in the inflamed tissues (reviewed in [5,10]). In mice, infDCs were initially identified as MHC II^+^ CD11b^+^ CD11c^+^ F4/80^+^ Ly6C^+^ [11]. However, these, as well as other markers later identified, are also expressed on other types of myeloid cells such as macrophages and monocytes. Therefore, a functional assay demonstrating the ability to activate T cells is normally required to definitively identify infDCs. When their cellular origin was investigated, infDCs were found to differentiate from CCR2-expressing inflammatory monocytes that are characterized as CD11b^+^Ly6C^hi^. These cells are recruited to sites of inflammation and in a process that requires GM-CSF, and potentially other factors, differentiate into infDCs [5]. However, depending on the model system under investigation, diverse functions have been assigned to the CCR2-expressing CD11b^+^Ly6C^hi^ cells or their differentiation products. These functions include control of microbes, immuno-pathology, immuno-stimulation and immuno-suppression [12,13]. In bacterial infection, the CCR2-expressing CD11b^+^Ly6C^hi^ cells (and their differentiation products) are generally required to control infection by direct killing of bacteria [13]. During *Listeria monocytogenes* infection, the CCR2-expressing Ly6C^hi^ cells produced large amounts of TNFα and iNOS, and were hence termed Tip-DC [14]. Even though TipDCs were able to stimulate alloreactive T cells in vitro, they were not required to induce an LLO_91-99_ specific CD8^+^ T cell response in mice infected with *L*. *monocytogenes*. In fact, the CD8^+^ T cell responses to the LLO_91-99_ epitope in the spleens of Ccr2^-/-^ mice infected with *L*. *monocytogenes* were larger than that observed in the corresponding spleens of wild type mice [14]. This could be due to the T cell-suppressive effect of the nitric oxide (NO) produced from these cells. In our previous study using a mouse model of *Salmonella enterica* serovar Typhimurium infection, recruitment of CD11b^+^Ly6C^hi^ cells to infected spleens also depended on CCR2, and these cells remained in an immature state in vivo, but could be differentiated further in vitro to express higher levels of MHC II and F4/80. Furthermore, these immature CD11b^+^Ly6C^hi^ cells also inhibited both CD4^+^ and CD8^+^ T cell proliferation via a NO-dependent mechanism in vitro [15]. Therefore, during bacterial infection of mice, CCR2-expressing CD11b^+^Ly6C^hi^ cells can acquire DC-like characteristics and have direct antimicrobial activity, but it is unclear if these cells can differentiate into infDC and prime or activate CD8^+^ T cell responses to microbial antigens.

A number of Gram-negative bacterial pathogens utilize type III secretion systems (T3SS) to inject effector proteins directly into the cytosol of infected host cells in order to overcome barriers to infection or counteract innate immune responses [16]. A well-studied T3SS that is required for virulence is encoded on a plasmid (pYV) in the enteric pathogen *Yersinia pseudotuberculosis*. From the pathogen’s viewpoint an unintended consequence of the T3SS process is that translocated effector proteins can serve as antigen for presentation by the classical class I pathway [17]. We recently showed that during primary infection of C57BL/6 mice with *Y*. *pseudotuberculosis*, an exceptionally large CD8^+^ T cell response is induced against the T3SS effector YopE. We consider this response as exceptionally large, because at the peak of the response, up to 50% of total CD8^+^ T cells in spleens are specific for H2-K^b^ class I MHC-restricted epitope YopE_69-77_ [18]. In comparison, during primary *L*. *monocytogenes* infection in mice, only 2–3% of splenic CD8^+^ T cells are specific for LLO_91-99_ at the peak of response [19], and even at the peak of a recall response, only ~17% of all CD8^+^ T cells in the spleen recognize LLO_91–99_ [20]. Following intragastric infection of C57BL/6 mice with *Y*. *pseudotuberculosis* a large YopE_69-77_-specific CD8^+^ T (ET) cell response is also detected in intestinal epithelia, lamina propria, and mesenteric lymph nodes [18,21]. ET cells elicited by vaccination with YopE_69-77_ peptide can protect against *Y*. *pseudotuberculosis* and the related pathogen *Yersinia pestis* through secretion of the cytokines TNFα and IFNγ [18,22,23]. The epitope YopE_69-77_ is located in the N-terminal chaperone-binding (Cb) domain of the effector. The C-terminal half of YopE contains the GTPase-activating protein (GAP) activity that is important for *Yersinia* virulence. GAP catalytic activity, as well as other important molecular characteristics of YopE, including its ability to localize to membranes or to undergo ubiquitination, is not required for the large ET cell response [24]. Factors important for the large ET cell response on the host side of the interaction are unknown, however we did observe that the number of ET cells positively correlated with the number of CD11b^+^ cells in the spleens of *Y*. *pseudotuberculosis-*infected mice [24].

Given the unprecedented magnitude of the ET cell expansion in *Y*. *pseudotuberculosis-*infected mice, it is important to further clarify the bacterial and host factors that are important determinants of this immune response. Here we show that production of the large ET cell response depends on T3SS-mediated translocation of YopE as well as infDCs, whose recruitment from bone marrow requires CCR2. In addition, in the absence of the large response to translocated YopE, we detected a compensatory adaptive immune mechanism in which secreted YopE appears to be cross-presented by CD8α^+^ DCs.

## Results

### Expansion of a diverse Vβ repertoire in ET cells during *Y*. *pseudotuberculosis* infection

**Fig 1:**
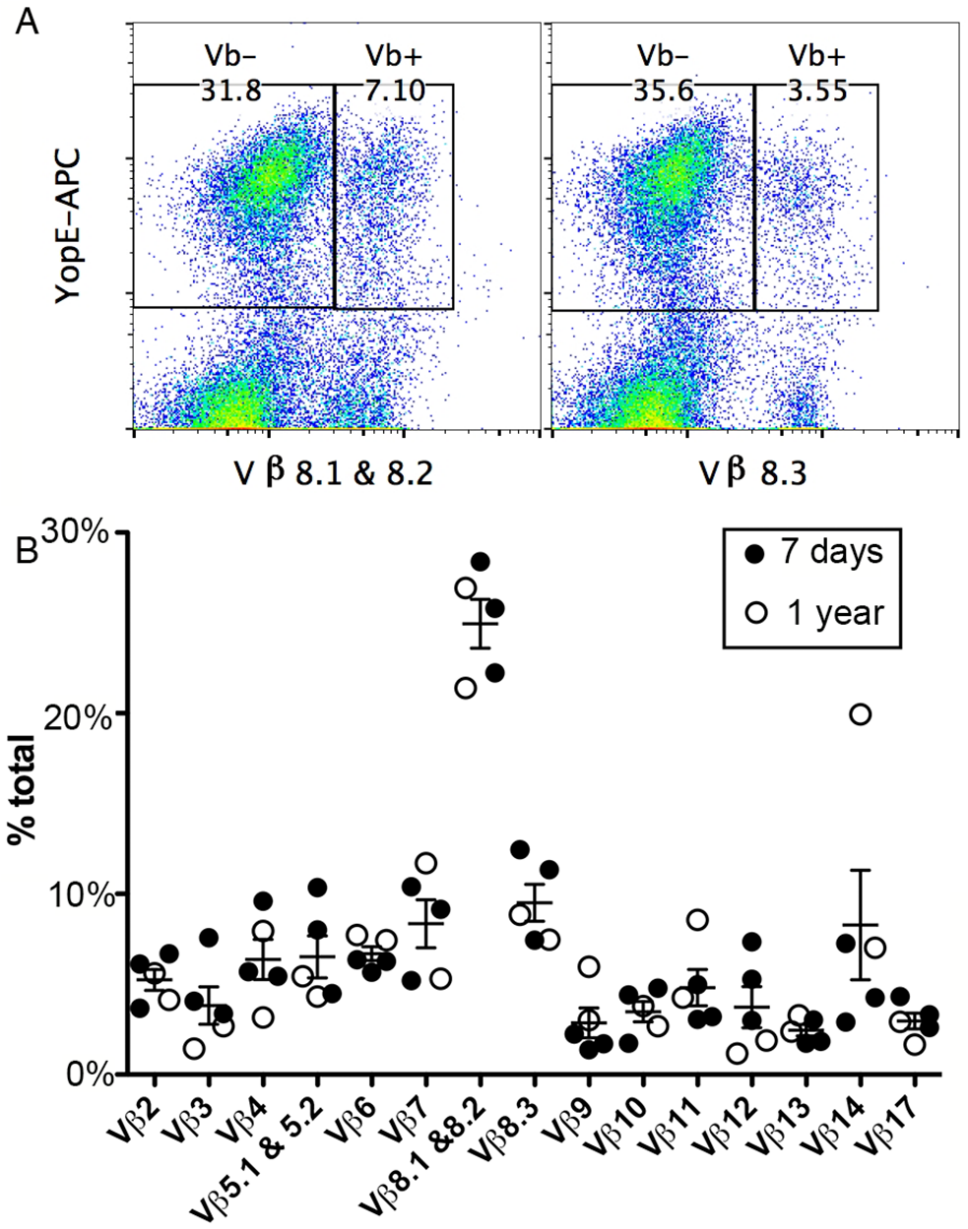
TCR Vβ subset distribution in ET cells. Splenocytes from C57BL/6 mice infected IV with *Y*. *pseudotuberculosis* mE for 7 days (A and filled circles in B) or one year (empty circles in B) were stained with YopE_69-77_ tetramer, a panel of Vβ antibodies and CD8 antibody conjugated with different fluorophores and were analyzed by flow cytometry. Representative histographs of tetramer (YopE-APC) and Vβ8.1 & 8.2 (left) or Vβ8.3 (right) signals from CD8^+^ T cells from one mouse are shown in (A). Numerical values correspond to percentages of gated cell populations among total CD8^+^ T cells. A summary of the percentages of each Vβ subset of total Vβ (% total) among ET cells from 3 mice infected for 7 days and 2 mice infected for 1 year is shown in (B). Mean and SEM is shown for each group of 5 mice.

The exceptionally large ET cell response in mice infected with *Y*. *pseudotuberculosis* is similar to the magnitude of CD4^+^ T cell responses to superantigens. Superantigens typically induce a CD4^+^ T cell response that is limited in diversity with respect to the Vβ usage in αβ T-cell receptors (TCRs). To determine the clonal nature of the ET cells produced during *Y*. *pseudotuberculosis* infection of C57BL/6 mice, the Vβ repertoire of these cells was investigated. The genes of functional TCRs are assembled from separate V, D, J region segments through recombination (reviewed in [25]). Mice and humans carry about 20–70 germline V segments that encode about 90 amino acid residues of the mature TCR. Therefore, diversity in Vβ composition demonstrates a polyclonal nature of a T cell population, however, T cells containing the same Vβ regions are further diversified through the addition of D and/or J segments and imprecise joining. To obtain uniform infections we used intravenous (IV) challenges, and because it is difficult to achieve sublethal infections via this route with the wild-type bacteria, our experiments were done with the attenuated *Y*. *pseudotuberculosis* strain 32777 encoding catalytically inactive YopER144A (mE, Table 1). C57BL/6 mice were infected IV with mE, and the Vβ composition of the ET cells in spleens was determined using a panel of fluorophore-conjugated antibodies recognizing different Vβ regions in conjunction with tetramer staining and flow cytometry (Fig 1A). Results obtained with an uninfected mouse analyzed in parallel as a control are shown in S1 Fig Seven days post infection (dpi), when the number and/or percentage of ET cells were still increasing; or one year after infection, when only ~2% of total splenic CD8^+^ T cells were specific for YopE_69-77_, the most prominent population was composed of Vβ8.1 and 8.2, with an average of 25% of all ET cells in this category (Fig 1A, left, and 1B). The 2^nd^ largest population, however, differed between individual mice (Fig 1B). Overall, all of the Vβ subsets tested were represented within the ET cell population in all the mice examined, ranging in average composition from 3% to 25% among all the ET cells (Fig 1B). These results revealed that the Vβ usage in the ET cell composition is polyclonal and highly diverse, indicating that an antigen-presentation process, rather than a superantigen-like mechanism, is responsible for production of these cells.

**Table 1:** *Y*. *pseudotuberculosis* strains used in the study.

Strain name	Relevant characteristic(s)	Reference or source
32777	Serogroup O:1, pYV^+^	[44]
mE	*yopE* ^*R144A*^, catalytically inactive allele of *yopE*	[44]
ΔB	*yopB40*, stop codon and frame shift in *yopB*	[44]
ΔBmE	*yopB40*, *yopE* ^*R144A*^	This study
ΔGAP	*yopE* ^*ΔGAP*^, deletion of codons corresponding to GAP domain	This study
ΔpYV	Cured of the virulence plasmid pYV	[45]
ΔYscF	*ΔyscF*, deletion of entire open reading frame	This study
YopE-Bla	Integration of plasmid into *yopE* in pYV generates translational fusion of chaperone-binding domain of YopE and TEM1 β-lactamase	This study
ΔB YopE-Bla	*yopB40*, integration of plasmid into *yopE* in pYV generates translational fusion of chaperone-binding domain of YopE and TEM1 β-lactamase	This study
YopEΔN15	*yopE* ^*Δ2–15*^, deletion of codons corresponding to signal sequence residues 2–15	This study
ΔYopE	*ΔyopE*, deletion of entire ORF	This study

### Construction and characterization of *Y*. *pseudotuberculosis* strains deficient for secretion and or translocation of YopE

**Fig 2:**
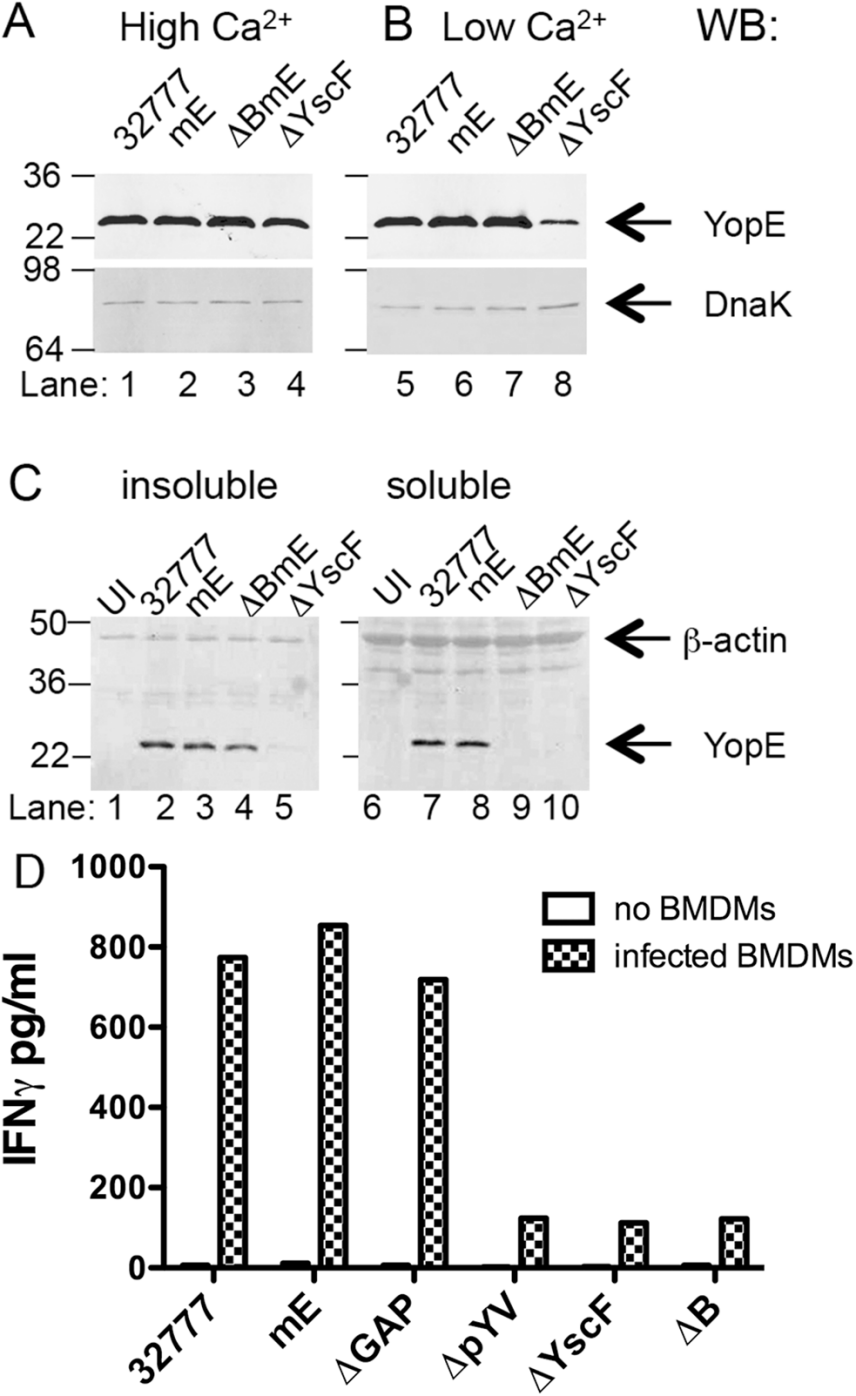
Measurement of production, translocation and antigen presentation of YopE in vitro. The indicated strains of *Y*. *pseudotuberculosis* were grown at 37°C in LB containing 2.5 mM of CaCl_2_ (High Ca^2+^) to inhibit type III secretion (A), or in LB containing 20 mM MgCl and 20 mM NaOX (Low Ca^2+^) to activate type III secretion (B). Lysates of the bacteria were subjected to immunoblotting with anti-YopE antibodies. Immunoblotting with anti-DnaK antibody was used to indicate equal loading. (C) The indicated strains grown at 37°C in low Ca^2+^ LB were used to infect BMDMs at a MOI of 50 for 1.5 h. The infected BMDMs were incubated with a non-iononic detergent buffer and then separated into an insoluble fraction containing bacteria or a soluble fraction containing cytosolic components. Samples of the insoluble (left panel) or soluble (right panel) fractions were subjected to immunoblotting with anti-YopE antibody or anti-β-actin antibody to control for loading. Results shown are representative of three independent experiments. (D) The indicated strains were added to wells without BMDMs or to wells containing BMDMs at MOI of 10 for 4 h, and gentamicin was included during the last 2 h. Then ET-enriched CD8^+^ T cell lines in medium containing penicillin and streptomycin were added to wells containing bacteria alone or to wells containing BMDMs infected with bacteria at 1:4 (BMDM to T cell) ratio and incubated for 48 h before measuring the concentration of IFNγ in the supernatants by ELISA. Data shown are results from one representative of 4 experiments performed.

To begin to identify bacterial and host factors required for the large ET cell response, we first focused on the pathogen and investigated whether secretion and translocation of YopE by the T3SS is required for induction of ET cells during infection. For this purpose, deletion mutations were introduced into *Y*. *pseudotuberculosis* 32777 to inactivate *yscF* or *yopB* (Table 1). The *yscF* mutant (ΔYscF) lacks the T3SS needle and is unable to secrete or translocate YopE. Deletion of *yopB* in 32777 or mE resulted in strains defective for translocation of YopE or YopER144A into host cell cytosol (ΔB and ΔBmE, respectively, Table 1). It is important to note that YopB is not required for effector secretion, and as a result YopE is released into the extracellular milieu during in vitro infection of host cells with a *Y*. *pseudotuberculosis yopB* mutant [26]. In bacterial growth media, steady state levels of YopE were similar in 32777, mE, and ΔBmE, either at high Ca^2+^ when YopE is produced but not secreted, or at low Ca^2+^ when YopE is produced and secreted, as determined by immunoblotting (Fig 2A and 2B, lanes 1–3, 5–7). Immunoblotting for DnaK was used to control for loading (Fig 2A and 2B). In contrast, steady state amounts of YopE were lower in ΔYscF especially in the low Ca2^+^ medium (Fig 2A and 2B, lanes 4 and 8). Next, the above strains were used to infect bone marrow-derived macrophages (BMDMs) from C57BL/6 mice and translocation of YopE was measured by detergent solubility and immunoblotting [27]. Immunoblotting for β-actin was used to control for loading. As compared to the control strains, ΔBmE failed to translocate YopE, as evidenced from its absence in the detergent soluble (cytosolic) fraction of infected BMDMs (Fig 2C, compare lanes 7–9). ΔYscF also did not translocate YopE (Fig 2C, lane 10), however, this strain also produced very low amounts of the protein during infection of BMDMs, as seen by analysis of the detergent insoluble (bacterial) fraction (Fig 2C, lane 5). These observations indicated that ΔBmE is selectively deficient in translocation of YopE while ΔYscF is deficient for production, secretion and translocation of YopE.

### YopE translocation is required for activation of ET cells in vitro

BMDMs infected with the *Y*. *pseudotuberculosis* strains described above were tested for to the ability to activate ET cells in vitro. In addition to 32777, mE, ΔYscF and ΔB, as controls we analyzed BMDMs infected with strains lacking the GAP domain of YopE (ΔGAP) or the virulence plasmid (ΔpYV) (Table 1). Co-culture of ET-enriched CD8^+^ T cell lines with *Y*. *pseudotuberculosis* inactivated with antibiotics in the absence of BMDMs, resulted in minimal IFNγ production (Fig 2D). In contrast, BMDMs infected with 32777, mE or ΔGAP supported secretion of IFNγ from co-cultured ET cells (Fig 2D). BMDMs infected with ΔpYV, ΔYscF or ΔB, did not support elevated secretion of IFNγ from ET cells (Fig 2D). These results indicate that translocation of YopE into the cytosol of BMDMs is required for activation of ET cells in vitro.

### Secreted and translocated YopE contribute differently to the large ET cell response in vivo

**Fig 3:**
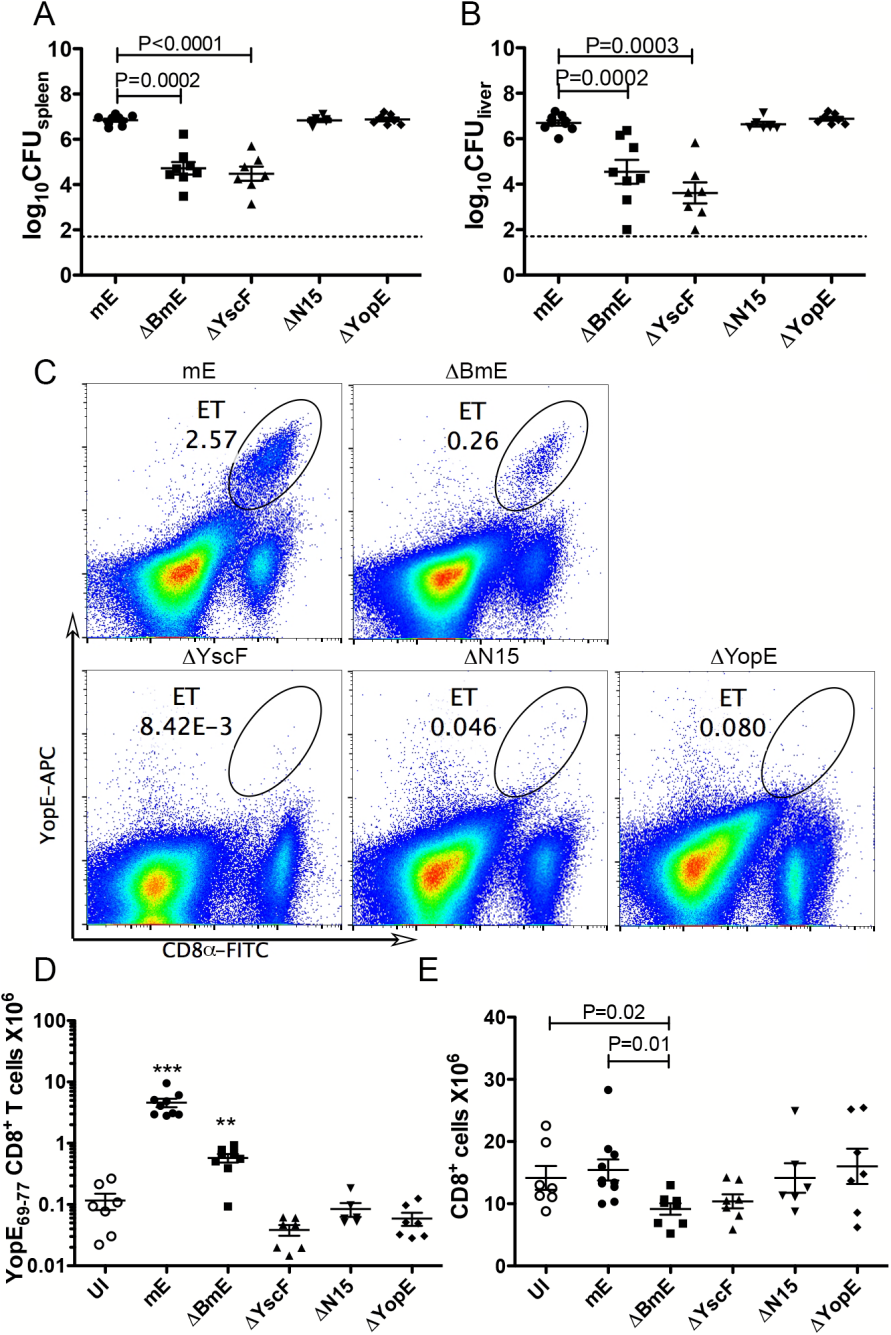
Translocation-dependent and-independent production of ET cells. Groups of C57BL/6 mice were infected IV with 1000 CFU of mE, 5X10^5^ CFU of ΔBmE, 1X10^5^ to 1X10^6^ CFU of ΔYscF or 1000 CFU of YopEΔN15 or ΔYopE or left uninfected (UI). The colonization levels in spleen (A) or liver (B) were determined by CFU assay 7 dpi. The detection limit of log_10_CFU = 1.7 is indicated with dotted line. (C) Representative histographs of CD8α and YopE_69-77_ tetramer signals on splenocytes from mice infected for 7 days with mE, ΔBmE, ΔYscF, YopEΔN15 or ΔYopE as indicated, with the percentage of ET cells in splenocytes indicated with oval gates. The number of splenic ET cells (D) and CD8^+^ T cells (E) in groups of mice at 7 dpi was determined by flow cytometry following tetramer and antibody staining. Each symbol represents the value obtained from one mouse. Data shown are the summary of two to four independent experiments. P values obtained with the Mann-Whitney test are shown when the values are statistically different between the indicated groups (A, B and E). In D, the values obtained from mice infected with mE or ΔBmE are significantly different from all others and are indicated with *** for P<0.001 and ** for P<0.01.

C57BL/6 mice were infected IV with mE, ΔYscF or ΔBmE to determine if secretion and translocation of YopE are required for the large ET response in vivo. It was expected that ΔBmE and ΔYscF would be more attenuated than mE, because the former strains are defective for translocation of all effectors, while mE is only missing the catalytic activity of YopE. To compensate for different levels of attenuation in the mutants, mice were infected with maximum sub-lethal doses of each strain. However, even with these adjusted doses, at 7 dpi, significantly lower levels of bacteria were recovered from the spleens and livers of mice infected with either ΔYscF or ΔBmE than those infected with mE (Fig 3A and 3B). Additional cohorts of mice infected as above with mE or ΔBmE were analyzed at 4 dpi and results showed that ΔBmE colonized spleen and liver at a significantly lower level than mE at this time as well (S2 Fig).

Next, the ET cell response was assessed at 7 dpi by tetramer staining and flow cytometry of splenocytes. As we have shown before [24], IV infection of mice with mE results in a large ET cell response in spleens where an average of 3.57 million of these cells are recovered at 7 dpi (Fig 3C and 3D). This ET cell number corresponds to ~30% of all CD8^+^ T cells in spleens of infected mice. Infection with ΔBmE resulted in a significantly reduced ET cell response both in cell number (average of 0.57 million) and percentage among the CD8^+^ T cells (average of 6.26%) comparing to those animals infected with mE (Fig 3C and 3D). These results suggested that the large ET cell response observed during *Y*. *pseudotuberculosis* infection requires translocation of YopE.

To confirm that the greatly decreased ET cell response in ΔBmE-infected mice was not due to significantly decreased bacterial load as compared to mE (Fig 3A and 3B), a new strain YopEΔN15 (Table 1) selectively defective for export of YopE was created by deleting the secretion signal in residues 2–15 of YopE [28]. Additionally, a strain lacking YopE (ΔYopE, Table 1) was constructed and used as a negative control. YopEΔN15 was defective for YopE translocation into infected BMDMs (S3 Fig). In mouse infection, YopEΔN15 colonized spleens and livers to the same extent as mE and ΔYopE (Fig 3A and 3B), yet the number of ET cells in spleens didn’t increase beyond that seen in mice left uninfected or infected with ΔYopE (Fig 3C and 3D). Therefore, YopE translocation is required for the large ET cell response.

The ET cell response in spleens of mice infected with ΔBmE was significantly higher than that in mice infected withΔYscF or YopEΔN15 (Fig 3C and 3D). However, similar numbers of total splenic CD8^+^ T cells were observed in mice infected with ΔBmE, ΔYscF or YopEΔN15 (Fig 3E). Thus, in the absence of the large translocation-dependent response, a lower but still significant “translocation-independent” ET cell response was detected in mice infected with the ΔBmE mutant but not the ΔYscF or YopEΔN15 mutants.

### The translocation-independent ET cell response is diminished in Batf3^-/-^ mice

**Fig 4:**
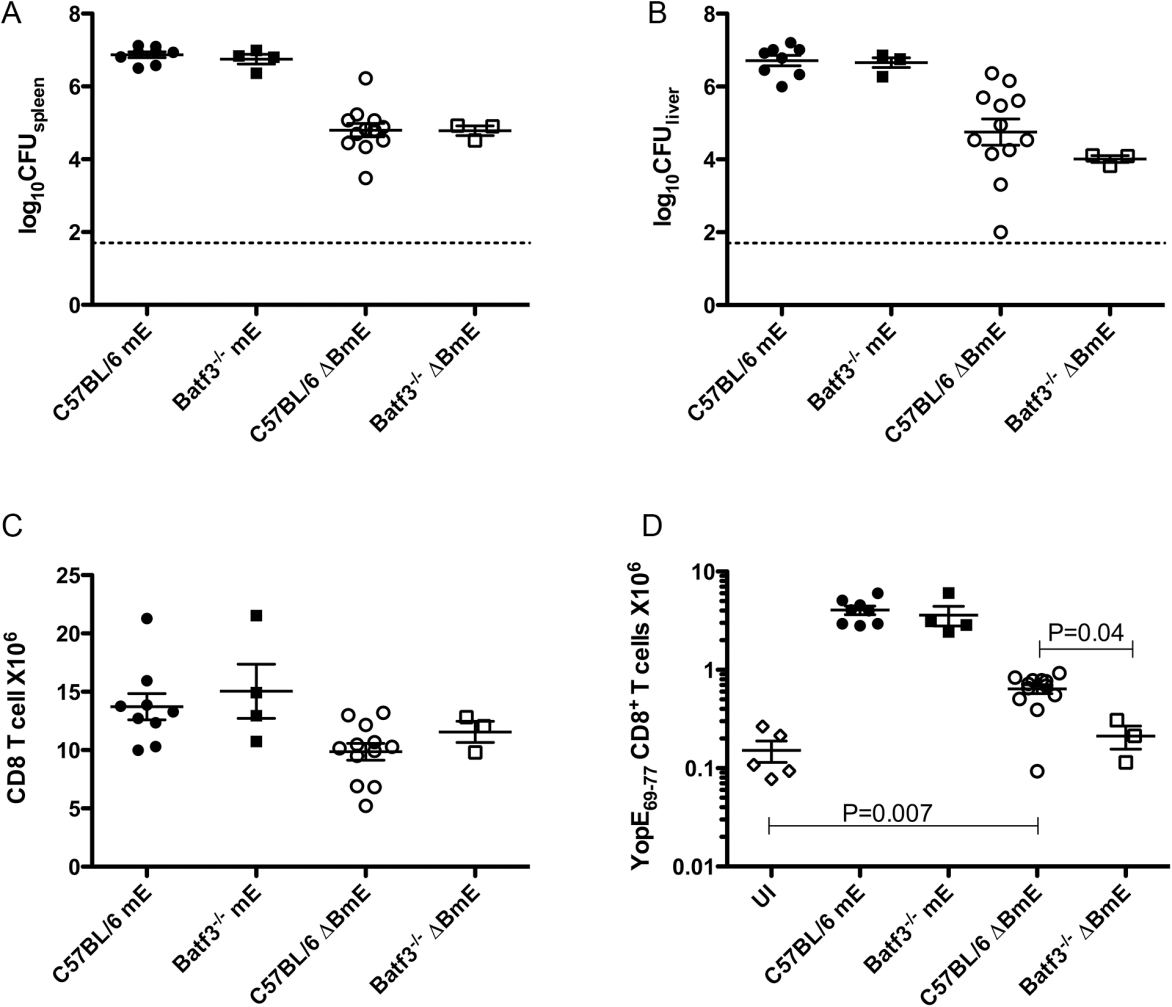
Diminished translocation-independent production of ET cells in Batf3^-/-^ mice. C57BL/6 or C57BL/6 Batf3^-/-^ mice were left uninfected (UI) or infected IV with 1000 CFU of mE or 5X10^5^ CFU of ΔBmE. Spleen (A) and liver (B) colonization levels were determined by CFU assay on 7 dpi. The number of splenic CD8^+^ T cells (C) and ET cells (D) was determined by flow cytometry following tetramer and CD8α staining. Data shown are the summary of two or more independent experiments. Non-colonized mice were removed from analysis and P values were determined with the Mann-Whitney test.

The translocation-independent ET cell response detected in mice infected with ΔBmE suggested the possibility that secreted YopE proteins were subject to cross presentation. Batf3^-/-^ mice deficient in the Batf3 transcription factor, lack CD8α^+^ DCs and the developmentally related CD103^+^ DCs and are thus defective in cross-presentation of extracellular proteins [8,9]. To study the role of cross presentation in the translocation-independent pathway, Batf3^-/-^ mice or C57BL/6 controls were infected IV with the maximal sub-lethal dose of mE or ΔBmE as above, followed by determination of bacterial numbers in organs, and numbers of CD8^+^ T cells and ET cells in spleen. At 7 dpi, comparable numbers of mE were recovered in spleens or livers of C57BL/6 and Batf3^-/-^ mice, and the same was true for ΔBmE (Fig 4A and 4B). Similarly, comparable numbers of CD8^+^ T cells were recovered from the spleens of the C57BL/6 and Batf3^-/-^ mice infected with mE or ΔBmE (Fig 4C). Furthermore, the ET cell numbers recovered from either C57BL/6 or Batf3^-/-^ mice infected with mE were comparable (Fig 4D). However, the number of ET cells in C57BL/6 mice infected with ΔBmE was significantly higher than in Batf3^-/-^ mice or uninfected C57BL/6 mice (Fig 4D). The number of ET cells in the spleens of Batf3^-/-^ mice infected with ΔBmE was in fact not different from that of C57BL/6 mice left uninfected. Collectively, these results indicate that in the absence of YopE translocation, cross presentation of secreted YopE can occur, leading to a smaller, yet still significant ET cell response.

### The large ET cell response to translocated YopE is significantly reduced in Ccr2^-/-^ mice

**Fig 5:**
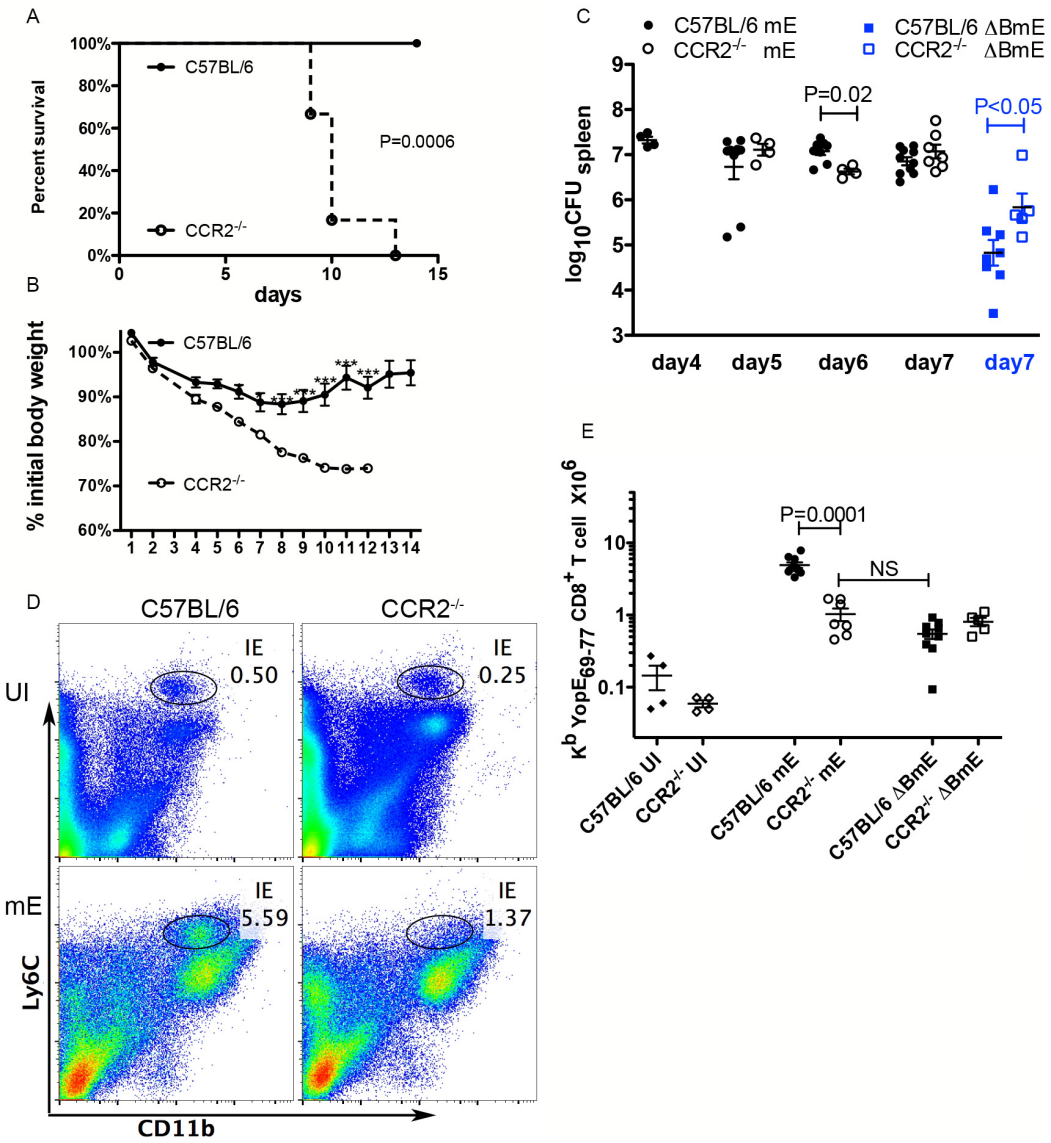
CCR2 is required for host survival and the large translocation-dependent ET cell response. Age and sex-matched C57BL/6 (filled symbols) or C57BL/6 Ccr2^-/-^ mice (open symbols) were left uninfected or infected IV with 1000 CFU of mE (circles) or 5X10^5^ CFU of ΔBmE (squares). Mouse survival (A) and weight (B) following infection with mE were monitored for 14 days. Data shown in (A) and (B) are from 1 experiment with 6 mice in each condition. Difference in survival was significant as determined with Log rank test. The differences in weight between the two groups of mice at different days were determined with two-way ANOVA followed by Bonferroni posttest. *, P<0.05; ***, P<0.001. (C) At different dpi as indicated with mE or 7 dpi with ΔBmE, spleen colonization levels were determined by CFU assay. (D) Representative histographs of Ly6C and CD11b signals on splenocytes from uninfected (UI) or mE-infected C57BL/6 or Ccr2^-/-^ mice at 7 dpi, and the CD11b^+^Ly6C^hi^ cells are indicated with an oval gate (percentage of total splenocytes within gate is shown). (E) The number of ET cells at 7 dpi was determined by flow cytometry following tetramer staining. Data shown in (C) and (E) are the summary of several independent experiments with at least two experiments at each time point. P values were determined with Mann-Whitney test as indicated in (C) or (E).

Our previous results demonstrated a linear correlation between the number of ET cells and the number of CD11b^+^ cells in the spleens of *Y*. *pseudotuberculosis-*infected mice [24]. CD11b^+^ cells recruited to infected tissues are a heterogeneous population of cells that include CD11b^+^Ly6C^hi^ inflammatory monocytes. These cells express CCR2, a chemokine receptor that promotes their emigration from the bone marrow [29]. Inflammatory monocytes can differentiate into infDCs, which contribute to host protection by presenting antigen to T cells [10]. To begin to investigate the role of infDCs derived from CCR2-expressing CD11b^+^Ly6C^hi^ cells in the dominant ET cell response, we infected Ccr2^-/-^ or C57BL/6 control mice IV with mE, and measured several parameters of the infection and immune response (mouse survival and weight, bacterial CFU and numbers of CD11b^+^Ly6C^hi^ and ET cells in spleens). With a dose of 1000 CFU, all wild type C57BL/6 mice survived infection to at least 14 days, and their body weights gradually decreased until 7–8 dpi, then recovered (Fig 5A and 5B). In contrast, the Ccr2^-/-^ mice lost body weight faster than the age-matched wild type C57BL/6 mice and the difference in weight became significant after 6 dpi (Fig 5B). Infected Ccr2^-/-^ mice also became obviously lethargic at 7 dpi, and died between 9–13 days (Fig 5A). From 5 to 7 dpi, the spleen colonization levels of mE in C57BL/6 and Ccr2^-/-^ mice were similar, with the exception that at 6 dpi bacterial numbers were significantly lower in Ccr2^-/-^ mice (Fig 5C). As expected, the accumulation of CD11b^+^Ly6C^hi^ cells observed in the spleens of C57BL/6 mice at 7 dpi was diminished in Ccr2^-/-^ mice (Fig 5D).

Time course analysis of the ET cell response in spleens showed that the numbers of these cells increased between 5 and 7 dpi in both C57BL/6 and Ccr2^-/-^ mice infected with mE (S4 Fig). However, at 7 dpi the number (Fig 5E) of ET cells were significantly lower in Ccr2^-/-^ mice than C57BL/6 mice. Comparable levels of ET cells were observed in the two groups of mice left uninfected (Fig 5E). When infection was carried out with the translocation-deficient strain ΔBmE, similar numbers of ET cells were present in C57BL/6 and Ccr2^-/-^ mice (Fig 5E), even though the two groups of mice were colonized to different levels by this strain (Fig 5C). Additionally, the numbers of ET cells in Ccr2^-/-^ mice infected with mE was not significantly different from the level seen in C57BL/6 or Ccr2^-/-^ mice infected with ΔBmE (Fig 5E). Overall, these results indicated an important role for CCR2-expressing CD11b^+^Ly6C^hi^ cells in the formation of the large ET cell response during *Y*. *pseudotuberculosis* infection. Based on these findings and additional experiments discussed below, we conclude that the CCR2-expressing CD11b^+^Ly6C^hi^ cells that are orchestrating the large translocation-dependent ET cell response are equivalent to infDC, and hereafter use this terminology to refer to this cell population.

### YopE is translocated into infDCs during *Y*. *pseudotuberculosis* infection

The TEM1 β-lactamase reporter has been used to identify cells that are injected with YopE in mice infected with *Yersinia* [30,31], however, it has not been shown that infDCs are targeted for YopE translocation. Therefore, we set out to monitor YopE translocation into infDC using the TEM1 β-lactamase-based fluorescence system.

A 32777 strain encoding the chaperone-binding domain of YopE fused to the TEM1 β-lactamase (YopE-TEM1) was created (YopE-Bla; Table 1). Upon incubation of splenocytes containing translocated YopE-TEM1 with the substrate CCF4-AM, intracellular β-lactamase will cleave the substrate causing the cell to fluoresce blue. It was determined that as few as ~100 molecules of β-lactamase can be detected in a single cell [32].

**Fig 6:**
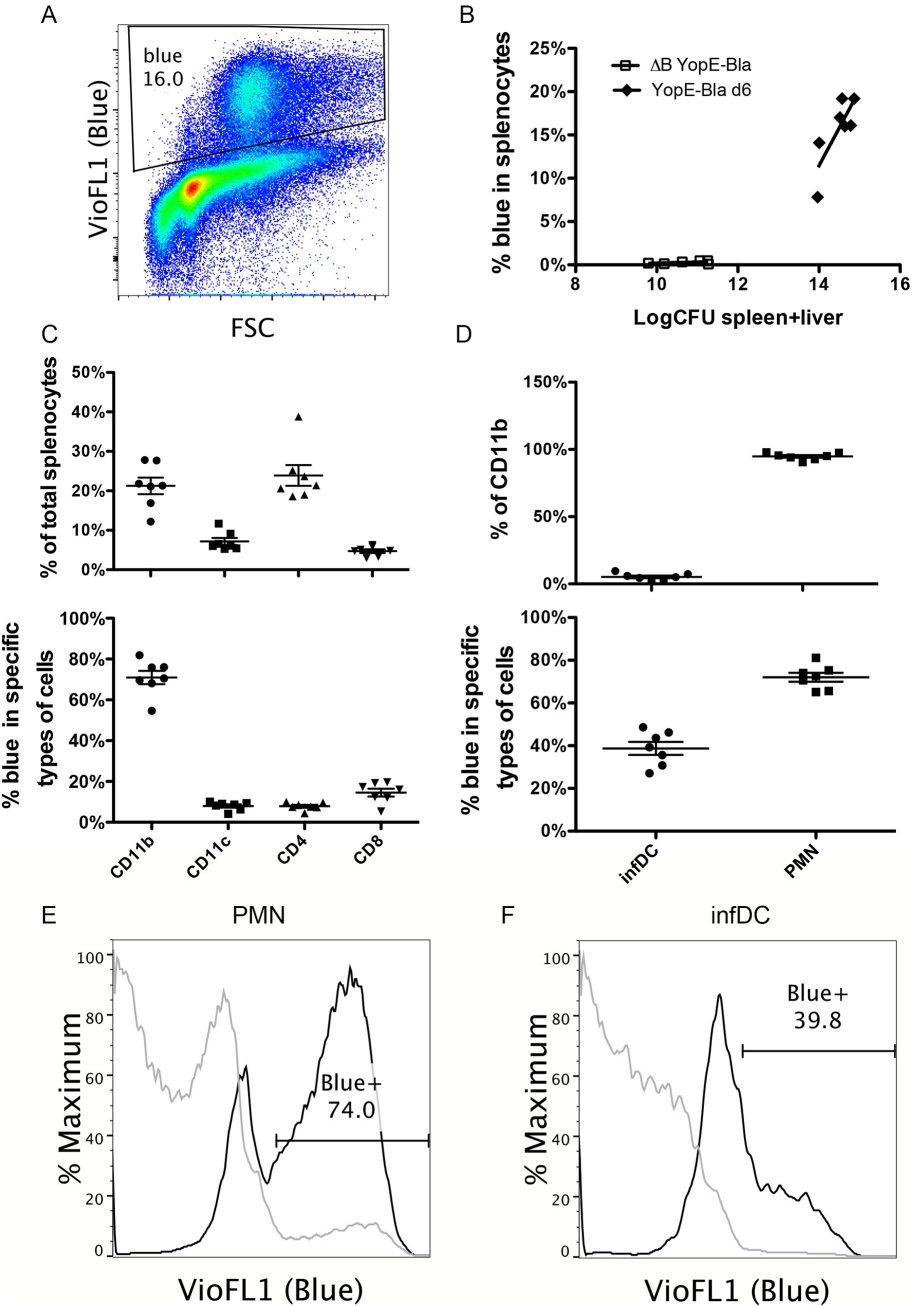
Detection of YopE-TEM1 translocation into different splenocyte populations. C57BL/6 mice were IV infected with 10^5^ CFU of YopE-Bla or 5X10^6^ CFU of ΔB YopE-Bla for 6 days. Splenocytes isolated from surviving mice were incubated with CCF4-AM to detect the presence of translocated YopE-TEM1, and subsequently stained with a panel of antibodies and analyzed with flow cytometry. (A) A representative histograph of splenocytes from a mouse infected with YopE-Bla showing the gate for translocation positive cells (Blue). (B) Spleen and liver colonization levels were determined by CFU assay for YopE-Bla, and ΔB YopE-Bla and the combined value of spleen and liver colonization was plotted against the percentage of blue splenocytes in the same mice. Data shown are the summary of four independent experiments. (C) Splenocytes from mice infected with YopE-Bla for 6 days were analyzed by flow cytometry and the percentage of splenocytes positive for the indicated markers (top) and blue cells among these cells (bottom) were determined. (D) Percentage of Ly6C^hi^ infDCs or Ly6C^med^ PMNs among the CD11b^+^ splenocytes from mice infected with YopE-Bla for 6 days were determined by flow cytometry (top), and the percentage of blue cells among these were plotted at bottom. Representative histograph of overlaid blue signal strength of PMNs (E) or infDC (F) from an individual mouse infected for 6 days with YopE-Bla (black lines) or ΔB YopE-Bla (light gray lines). The percentage of blue cells from the YopE-Bla-infected sample was indicated in the gate. In B-D, each dot represents the value obtained from one mouse, and wherever applicable, the bar indicated the average.

YopE-Bla was attenuated in comparison to mE in our IV mouse infection model, and therefore an infection dose of 10^5^ CFU was used. At 6 dpi of C57BL/6 mice with YopE-Bla, approximately 16% of splenocytes were blue as a result of translocation of the YopE-TEM1 fusion protein (Fig 6A). Translocation of YopE-TEM1 increased with increasing colonization levels of YopE-Bla in spleen and liver (Fig 6B). Translocation largely depended on YopB because an average of only 0.5% of the total splenocytes were blue after infection with the control ΔB YopE-Bla strain (Table 1) (Fig 6B).

Flow cytometry was used to quantify the percentage of CD11b^+^, CD11c^+^, CD4^+^ or CD8^+^ cells in splenocytes, and the percentage of these cells that were blue as a result of YopE-TEM1 translocation at 6 dpi with YopE-Bla. Consistent with our previous observation [24,33], a large percentage (~21%) of total viable splenocytes were identified to be CD11b^+^ (Fig 6C top). Approximately 20% of splenocytes were CD4^+^, while lower numbers (<10%) of splenocytes were CD11c^+^ or CD8^+^ (Fig 6C, top). Among the CD11b^+^ cells, an average of 71% were blue as a result of YopE-TEM1 translocation (Fig 6C bottom). In contrast, ~8% of CD11c^+^ or CD4^+^ cells or 14% of the CD8^+^ T cells, were blue as a result of YopE-TEM1 translocation (Fig 6C, bottom).

Next, we determined the numbers of CD11b^+^ cells that were Ly6C^hi^ or Ly6C^med^, considering the former infDCs and the later PMNs, and quantified the percentages of these cells that were subject to YopE-TEM1 translocation. After infection with of YopE-Bla, ~5% of all CD11b^+^ cells were Ly6C^hi^ infDC and the remainder were Ly6C^med^ PMNs (Fig 6D, top). This skewed increase in the percentage of PMNs most likely reflected a heightened yet unproductive inflammatory response in the mice terminally infected with YopE-Bla. Consistent with previous studies where PMNs represented the major recipients of translocated YopE [30], a greater percentage of PMNs (~72%) than infDCs (~39%) were blue in mice infected with YopE-Bla (Fig 6D, bottom, 6E and 6F, black lines). Smaller percentages of PMNs (~5%) and infDCs (~0.75%) were blue in mice infected with ΔB YopE-Bla (Fig 6E and 6F, gray lines), confirming that translocation of YopE-TEM1 in vivo was largely YopB dependent. These results indicated that infDCs were subject to YopE translocation during *Y*. *pseudotuberculosis* infection, albeit to a lesser degree than PMNs.

### CCR2-expressing infDCs from *Y*. *pseudotuberculosis-*infected mice activate ET cells ex vivo and increase the percentage of ET cells among the CD8^+^ T cells after adoptive transfer into infected Ccr2^-/-^ mice

**Fig 7:**
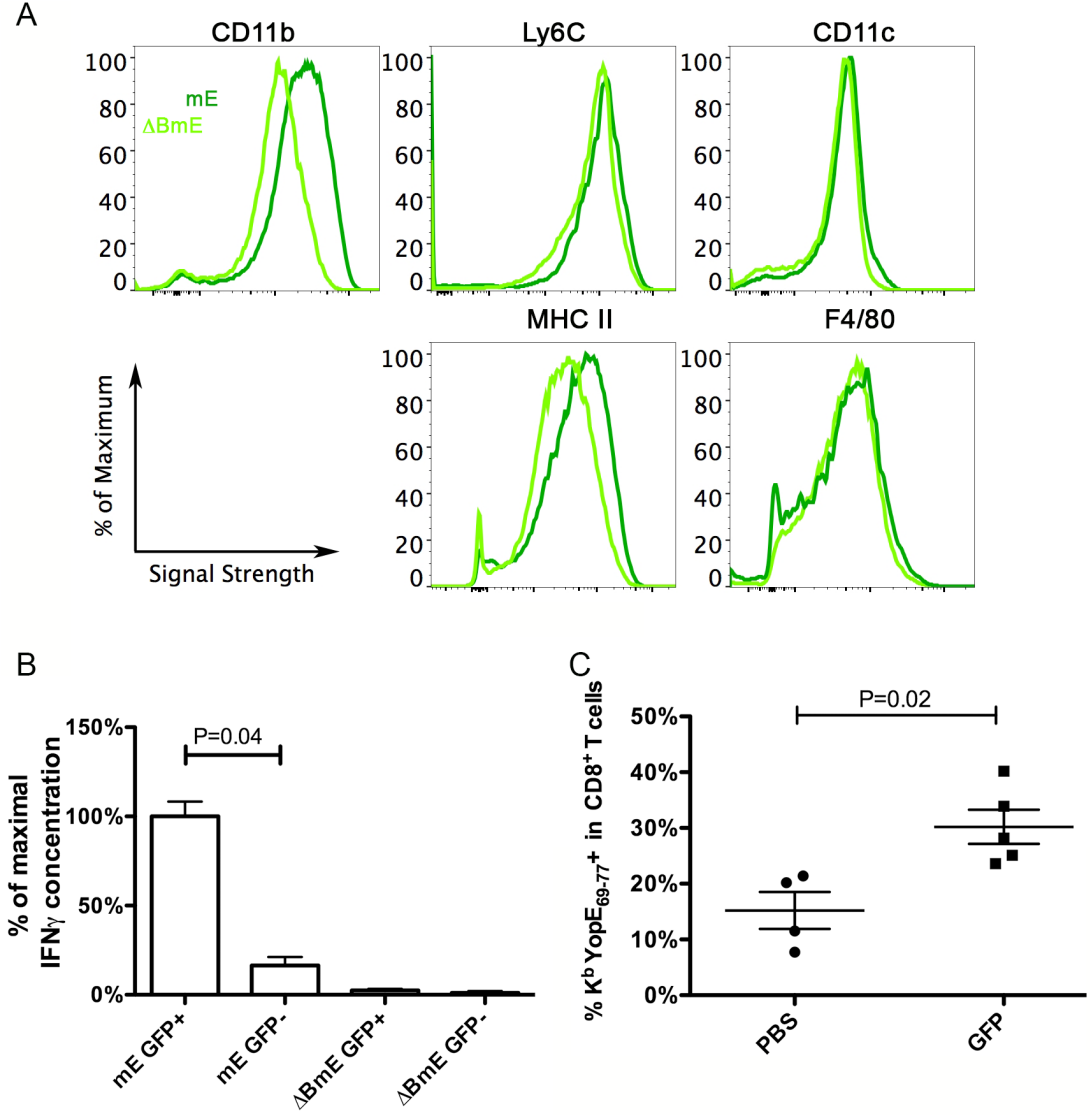
Phenotypic characterization of CCR2-expressing infDCs and their analysis by DEAD assay and adoptive transfer. (A and B) CCR2-GFP mice were infected IV with 1000 CFU of mE or 5X10^5^ CFU of ΔBmE, and 7 days later, total monocytes from spleens were isolated by negative selection. GFP^+^ cells were sorted from the GFP^-^ cells and characterized using flow cytometry (A, dark and light green lines representing cells from mice infected with mE orΔBmE, respectively). Results shown are representative of two independent experiments performed. After incubating the indicated GFP^+^ or GFP^-^ subpopulations with ET-enriched CD8^+^ T cells for 48 h, the concentrations of IFNγ were determined (B). The average concentration of IFNγ from samples obtained with GPF^+^ cells isolated from mE-infected mice was set at 100%, and the other values were normalized accordingly within the same experiment. The results shown in (B) are a summary of two experiments performed with a total of 3 mice infected with mE and 2 mice infected with ΔBmE. P value was determined with one way ANOVA (Kruskal-Wallis Test) followed by Dunn’s Multiple Comparison Test. (C) Four days post IV infection of CCR2-GFP mice with 1000 CFU of ΔYopE, GFP^+^ cells were enriched and sorted. PBS (PBS) or 1.8-2X10^6^ GFP^+^ (GFP) cells were injected to Ccr2^-/-^ mice that were infected with 1000 CFU of mE the day before. Six days later or 7 dpi, the percentages of ET cells among all CD8^+^ T cells were determined. By Mann-Whitney test, P = 0.02. Each symbol represented the value obtained from one mouse and the results shown are the summary of three independent experiments.

Next, we sought to further characterize the CCR2-expressing Ly6C^hi^ infDCs that are present in *Y*. *pseudotuberculosis-*infected mouse spleens, and to determine if these cells can directly activate ET cells. CCR2 reporter mice, which express enhanced GFP under the control of the murine CCR2 promoter [34], were infected as above with mE or ΔBmE. At 7 dpi, the average spleen colonization of mice infected with mE was 10^6.9^ CFU, while that of mice infected with ΔBmE was 10^5.1^ CFU, comparable to results in wild type mice infected with these strains of *Y*. *pseudotuberculosis*. After enrichment of splenic monocytes through negative selection, the GFP^+^ cells were isolated from GFP^-^ cells by sorting and phenotypically characterized by flow cytometry before they were used in direct ex vivo antigen display (DEAD) assays [35]. The GFP^+^ cells sorted from mice infected with mE expressed high levels of CD11b and Ly6C (Fig 7A, dark green lines). In addition, these cells also expressed intermediate levels of CD11c, MHC class II and F4/80 (Fig 7A, dark green lines). In comparison, the GFP^+^ cells isolated from mice infected with ΔBmE expressed these surface markers as well, though the levels of CD11b and MHC class II were lower (Fig 7A, light green lines). Thus, the GFP^+^ cells from mice infected with mE were phenotypically in line with the characteristics of infDCs [10].

The isolated GFP^+^ cells characterize above were tested for the ability to activate ET-enriched CD8^+^ T cell lines using DEAD assay [35]. GFP^-^ cells were analyzed in parallel as a control. Significantly higher amounts of IFNγ were produced by the ET-enriched CD8^+^ T cell lines when they were co-cultured with GFP^+^ cells from mE-infected mice, as compared to the GFP^-^ cells from the same mice or either cell type from ΔBmE-infected mice (Fig 7B). These results indicated that CCR2-expressing infDC that are isolated from *Y*. *pseudotuberculosis* mE-infected spleens can activate ET cells ex vivo.

Adoptive transfer experiments were carried out to determine if an ET response could be reconstituted in Ccr2^-/-^ mice by adoptive transfer of CCR2-expressing infDC. GFP^+^ cells were isolated as above from spleens of CCR2-GFP mice infected for 4 days with ΔYopE, to ensure that these cells do not carry the YopE_69-77_ antigen. The GFP^+^ cells were adoptively transferred into Ccr2^-/-^ mice that had been infected the day before with mE. Six days later, the percentages of ET cells among all CD8^+^ T cells in spleens were quantified by tetramer staining and flow cytometry. As shown in Fig 7C, the percentage of ET cells was significantly higher after adoptive transfer of GFP^+^ cells as compared to treatment with PBS alone as a control. Thus, an ET response was reconstituted after adoptive transfer of CCR2-expressing infDC into Ccr2^-/-^ mice infected with mE.

## Discussion

Here we have provided evidence that infDCs and CD8α^+^ DCs contribute to the production of antigen-specific CD8^+^ T cells using differentially localized (secreted vs. translocated) YopE during *Y*. *pseudotuberculosis* infection. Our results indicate that CCR2-expressing infDCs derived from CD11b^+^Ly6C^hi^ cells are required to produce the majority of ET cells in a YopE antigen “translocation-dependent” pathway, while CD8α^+^ DCs cross-present YopE_69-77_ in a “translocation-independent” manner. During *Y*. *pseudotuberculosis* infection, the translocation-dependent pathway dominates and leads to the formation of an unusually large number of ET cells. The unique combined requirements of YopE antigen delivery by the *Yersinia* T3SS and CD8^+^ T cell activation by host infDCs, resulting in the formation of large ET cell response, represents a new mechanism to generate an antigen-specific CD8^+^ T cell response.

CCR2 was required for host protection during *Y*. *pseudotuberculosis* mE infection, and curiously, its function was more important in the adaptive response stage. With our infection dose, in both C57BL/6 and Ccr2^-/-^ mice, adaptive response was evident 5 dpi because ET cells started to be detectable at higher levels at this time than those in the mice left uninfected (S4 Fig). Yet in mE-infected Ccr2^-/-^ mice, mice began to succumb at 9 dpi (Fig 5A). Furthermore, although both C57BL/6 mice and Ccr2^-/-^ mice lost weight post infection, it was only until 6 dpi that a significant difference was observed (Fig 5B). More importantly, from 5 to 7 dpi, the average colonization levels in the spleens of Ccr2^-/-^ mice infected with mE were not higher than those of C57BL/6 mice (Fig 5C). These observations support the idea that CCR2 function was important for host protection during the adaptive response stage. These results are different from previous studies with *L*. *monocytogenes*. For example, depletion of CCR2-postive cells from CCR2-DTR mice resulted in death of the mice from *L*. *monocytogenes* infection at 3 dpi [36]. Although our data do not rule out that infDCs, or their precursors the inflammatory monocytes, participate in host protection through direct killing of *Y*. *pseudotuberculosis*, we favor the idea that infDCs exert their important host protective function through activating CD8^+^ T cells during infection.

The CCR2-dependent CD11b^+^Ly6C^hi^ cells in spleens at 7 dpi with *Y*. *pseudotuberculosis* mE were characterized as infDCs. These cells expressed MHC class II molecules, were positive for F4/80, and CD11b, and expressed medium levels of CD11c (Fig 7A). More importantly, these cells obtained from mE-infected spleens activated ET-enriched CD8^+^ T cell lines to secrete IFNγ ex vivo (Fig 7B), and stimulated production of ET cells upon adoptive transfer into Ccr2^-/-^ mice infected with mE (Fig 7C). InfDCs have been implicated before to be important in innate host protection against *Y*. *pestis* infection in mice, and interestingly the effector YopM was shown to inhibit their recruitment to spleens [37]. Here we obtained evidence that CCR2-expressing infDCs were required to generate the large translocation-dependent ET cell response in mice infected with *Y*. *pseudotuberculosis* (Fig 5E). In comparison, previous studies indicated that CCR2-expressing CD11b^+^Ly6C^hi^Ly6G^-^ cells recruited to tissues of mice infected with *S*. Typhimurium had features of immature myeloid cells that exhibited both protective and immunosuppressive properties through producing NO [15]. Upon ex vivo culture with OT-I or OT-II T cells the *S*. Typhimurium-induced immature myeloid cells were able to present OVA peptide, but at the same time inhibited the proliferation of the CD4^+^ or CD8^+^ T cells by an NO-dependent mechanism [15]. Furthermore, the infDCs characterized here are likely to be different from the Tip-DCs described during *L*. *monocytogenes* infection [14]. In the absence of the infDCs in Ccr2^-/-^ mice infected with mE, there was a ~10-fold decrease in the number of ET cells recovered from the spleens of the infected animals (Fig 5E). In contrast, even though Tip-DCs could prime naïve alloreactive T cells in vitro, in their absence in the spleens of the *L*. *monocytogenes*-infected Ccr2^-/-^ mice, the numbers of the LLO_91-99_ specific CD8^+^ T cell were actually larger than that in the wild type mice similarly infected. This potentially suppressive effect was also likely due to the NO produced by the Tip-DCs [14]. These findings suggest that distinct features of *Y*. *pseudotuberculosis* pathogenesis and the resulting host response leads to the CCR2-dependent production of infDCs, which are functionally different from the CD11b^+^Ly6C^hi^Ly6G^-^ cells that are recruited to tissues of mice infected with *S*. Typhimurium (i.e. immature myeloid cells) or *L*. *monocytogenes* (i.e. Tip-DCs). Understanding the unique features of *Y*. *pseudotuberculosis-*host interactions that lead to the production of infDCs may have an impact on future design of vaccines to prime antigen-specific CD8^+^ T cell responses.

There are at least two possible pathways by which infDCs promote the formation of the large ET cell response. First, the infDCs may activate the ET cells in an antigen-dependent manner. As we have shown here, these cells were subjected to the T3SS-mediated injection of YopE from *Y*. *pseudotuberculosis* (Fig 6). The cytoplasmic location of YopE presumably allowed the antigenic peptide YopE_69-77_ to be presented through the classical pathway as has been proposed before [38]. Alternatively, infDCs (or their inflammatory monocyte precursors) may activate the ET cells indirectly through secretion of cytokines such as IL-18. It has been shown that inflammatory monocytes activate both NK cells and memory CD8^+^ T cells through producing IL-18 and IL-15 during infection [39]. IL-18 has been shown to be required for host protection during *Yersinia* infection [40–42]. In addition, elevated levels of IL-18 were detected in serum during *Y*. *pseudotuberculosis* infection [33,43]. It is possible that infDCs activate ET cells through producing IL-18, as well as presentation of the YopE_69-77_ peptide. The diverse TCR Vβ usage of the ET cells (Fig 1) is suggestive of multiple independent antigen-presenting events. The levels of ET cells observed in the Batf3^-/-^ mice infected with mE were comparable to that observed in wild type mice infected with mE (Fig 4E), indicating that when the translocation-dependent pathway is operating CD8α^+^ DCs are not required to present YopE peptide to CD8^+^ T cells. CD8α^+^ DCs were only required to cross present YopE peptide if infection was carried out with strain ΔBmE, since the levels of ET cells in the Batf3^-/-^ mice infected with ΔBmE decreased to the levels seen in mice left uninfected (Fig 4E). Though we cannot rule out that other types of DCs present the antigenic epitope YopE_69-77_ to CD8^+^ T cells, we think it is possible that during *Y*. *pseudotuberculosis* infection, infDCs both present the antigen to the ET cells and activate them through secretion of cytokines such as IL-18. Future studies are needed to distinguish the relative contributions of these two activities to the production of the large ET response.

Another interesting finding here is the requirement of CD8α^+^ DCs, in eliciting the ET cells by a “translocation-independent” pathway during infection with a *Y*. *pseudotuberculosis yopB* mutant. CD8α^+^ DCs are required for cross-presentation in vivo [8,9]. During infection of cultured cells in vitro, *Y*. *pseudotuberculosis* has been shown to secrete YopE into the surrounding environment, especially in the case of infection with a *yopB* mutant [26]. Our results here indicated that during infection with the *yopB* mutant, secreted YopE is taken up and cross-presented by the CD8α^+^ DCs in vivo. The *yopB* mutant ΔBmE was unable to translocate Yops into host cell cytosol, yet was competent to secrete Yops into its environment. In contrast, the mutant ΔYscF was incompetent in both secretion and translocation. Consistently, ΔBmE elicited a smaller yet significant ET response, while ΔYscF was totally deficient in producing an ET response (Fig 3D). To further strengthen the point that only secreted or translocated YopE are presented to CD8^+^ T cells, YopEΔN15 was incapable to secrete YopE, and infection with this strain resulted in similar levels of bacterial colonization in deep tissues (Fig 3A and 3B). Yet YopEΔN15 didn’t elicited ET cells beyond what was seen in mice left uninfected (Fig 3C and 3D). Cross-presentation appeared to be the sole pathway used to present the secreted YopE to CD8^+^ T cells during infection with ΔBmE, since ET cell numbers in Batf3^-/-^ mice infected with this mutant were similar to those in mice left uninfected (Fig 4D). Cross-presentation of YopE by CD8α^+^ DCs may have contributed to the formation of the large ET response during infection with the strain mE. In the Ccr2^-/-^ mice infected with mE, the number of ET cells did decrease dramatically, but the number is not lower than that in mice infected with ΔBmE (Fig 5E). This indicated that in the absence of infDCs as seen in the Ccr2^-/-^ mice, other cells-most likely the CD8α^+^ DCs, presented YopE_69-77_ to CD8^+^ T cells. Nevertheless, the fact that ΔBmE elicited ET cells in a CD8α^+^ DC-dependent manner suggested that the *yopB* mutant of *Y*. *pseudotuberculosis* could be used to specifically target antigens, by a translocation-independent mechanism, toward cross-presentation pathways to elicit class I MHC-restricted immune responses.

## Materials and Methods

### Ethics statement

Use of mice for the preparation of BMDMs and for infection experiments was carried out in accordance with a protocol that adhered to the Guide for the Care and Use of Laboratory Animals of the National Institutes of Health (NIH) and was reviewed and approved (approval #206152) by the Institutional Animal Care and Use Committee at Stony Brook University, which operates under Assurance #A3011-01, approved by the NIH Office of Laboratory Animal Welfare.

### Bacterial strains

The *Y*. *pseudotuberculosis* strains used in this study are derived from a serogroup O:1 strain 32777 (Table 1). The mutant strains mE (*yopER144A*), ΔB (*yopB40*), and ΔpYV (cured of the virulence plasmid) have been described [44,45]. The *yopER144A* mutation was introduced into ΔB to create strain ΔBmE using allelic exchange as described [44]. Additional mutant strains were generated by the same method after constructing appropriate allelic exchange vectors using the plasmid pSB890. The vector used to construct ΔYscF was created using PCR and primers 5'- CAATCATCAACGCTATCCAGAAGG -3' and 5'-(tctaga)TTCCCACCGCTTACCAAACGAG -3' to amplify the operon *yscDEFGH*. A vector containing this operon was subjected to QuickChange mutagenesis using primer 5’-CATTATGTAGCAGGAGACCTAAAATAAGCTTATGAAATATAAACTCAACGTACTGTTAGC -3’ and its reverse complement to delete the coding region of *yscF*.

To construct ΔGAP, YopEΔN15, and ΔYopE, pSB980 vectors encoding *yopE* and its 5’- and/or 3’-region were modified as follows. Primer 5’-CCGGTGGTGACACCAGCTGCATGATATGGATAAAAACAAGGGGA-3’ and its reverse complement were used in QuickChange mutagenesis to delete codons 89 to 219, which correspond to the RhoGAP domain. Primer 5’-AGCCAAGGTAATAAATAGTC ATGTCTGTGTCAGGATCTAGC-3’ and its reverse complement were used in QuickChange mutagenesis to delete codons 2–15, which correspond to the secretion signal. Primer 5’-GTTTTAATAGCCAAGGTAATAAATAGTCTGATATG GATAAAAACAAGGGG-3’ and its reverse complement primer were used to delete the entire coding region of *yopE*. To construct strains YopE-Bla and ΔB YopE-Bla expressing YopE-TEM1 fusion protein, a pSB980 vector encoding codons 1–86 of *yopE* fused in frame to the open reading frame of TEM1 β-lactamase was constructed. The resulting vector was conjugated into 32777 and ΔB, and integration of the plasmid into *yopE* on pYV by homologous recombination was selected for using the gene encoding tetracycline resistance on pSB890. Isolates generated from the desired integration events were identified by testing for T3SS-mediated secretion of the YopE-TEM1 fusion protein under low calcium growth conditions.

### Mice and infection conditions

Female C57BL/6J and B6.129S(C)-*Batf3* ^*tm1Kmm*^/J (stock Number 013755, Batf3^-/-^) mice were from Jackson Laboratory. CCR2-GFP mice and Ccr2^-/-^ mice on the C57BL/6 background (both provided by Dr. Eric Pamer) were bred at Stony Brook University. For intravenous (IV) infection, over night bacterial culture grown in Luria-Bertani (LB) at 28°C were washed once and re-suspended in phosphate buffered saline (PBS) to achieve the desired CFU/ml. Then 200 μl volumes were delivered via lateral tail vein. At indicated times post infection, or when death was imminent, mice were euthanized by CO_2_ asphyxiation. Mouse spleens and livers were dissected aseptically and weighed. Spleens were homogenized with a 5 ml syringe plunger in 5 ml of Dulbecco’s Modified Eagle Medium (DMEM). Livers were homogenized using a Stomacher80 (Seward Lab System) in 4 ml of PBS. Serial dilutions in LB were plated (100 μl) on LB agar to determine bacterial colonization by CFU assay, and the limit of detection was 50 CFU or log10 CFU of 1.7. All procedures for working with mice were approved by the Stony Brook University Institutional Animal Care and Use Committee.

### Immunoblotting analysis and detergent solubility assay

Two different growth conditions were used to prepare bacterial lysates. For the high calcium condition to encourage Yop synthesis but inhibit their secretion into medium, overnight cultures were diluted to OD600 of 0.1 into LB containing 2.5 mM of calcium chloride and grown at 37°C with shaking for 2 h. For the low calcium condition to encourage both synthesis and secretion of Yops, overnight cultures were diluted to OD600 of 0.1 into LB containing 20 mM of magnesium chloride and 20 mM of sodium oxalate and grown at 28°C for 1 h then 37°C for 2 h with shaking. After growth under one of the conditions above, the bacterial cultures were centrifuged, and the pelleted bacteria were resuspended in Hank’s Balanced Salt Solution (HBSS). After a second centrifugation, the pelleted bacteria were resuspended into 2X Laemmli sample buffer. To prepare secreted Yops, bacterial cultures in low calcium conditions were grown at 28°C for 1 h then 37°C for 4 h with shaking. Yop proteins in culture supernatants were precipitated with 10% trichloroacetate, washed once in cold acetone, dried and resuspended in 1X Laemmli sample buffer.

Detergent solubility assay was used to determine the amount of YopE translocated into the cytosol of BMDMs as described [24]. Briefly, bacteria were grown in the low calcium condition at 28°C for 1 h then 37°C for 2 h with shaking, washed and resuspended in HBSS. Then the bacteria were diluted into 1 ml of BMM-low medium and applied to C57BL/6L-derived BMDMs at 8X10^5^ cells/well on 6-well plate at MOI of 50. After incubating for 1.5 h, the monolayer was washed with PBS and scraped into 50 μl of 1% Triton X-100 buffer (10 mM Tris pH7.6, 150 mM NaCl, 10% glycerol, 1% Triton X-100) containing protease inhibitor cocktail (Roche). The lysate was centrifuged for 10 min at 12,000 g at 4°C to separate the supernatants (soluble fractions) from the pellet (insoluble fractions). The resulting supernatants of pellets were mixed with or resuspended in Laemmli sample buffer.

Bacterial lysates, Yop proteins and macrophage fractions were resolved by SDS-PAGE and transferred to nitrocellulose membrane, and analyzed by immunoblotting with mixture of monoclonal antibodies against YopE, or DnaK (clone 8E2/2; Stressgen) as described before [46].

### Flow cytometry

Single cell suspensions of spleens were prepared as described before [18]. Briefly, splenocytes in suspension were incubated in additional 20 ml of DMEM containing Penn/Strep for 20 m. Then red blood cells (RBC) were lysed, and viable cells were counted using typan blue exclusion with Countess (Invitrogen). Suspended cells (1X10^6^) were blocked using anti-mouse CD16/CD32 (FcgIII/II receptor) clone 2.4G2 (BD Pharmingen) and labeled with allophycocyanin-conjugated MHC class I tetramer K^b^YopE_69-77,_ which was provided by the NIH Tetramer Core Facility (Emory University, Atlanta, GA), at room temperature for 1 h and fluorophore-conjugated antibodies on ice for 20 minutes. The antibodies used were AlexaFluor488 or PE anti-mouse CD8α (53–6.7, BD, BioLegend), and PerCP anti-mouse CD3e (clone 145-2C11, PharMingen). CD8^+^ T cells were gated as CD3^+^CD8^+^ events throughout the study.

Vβ Screening was carried out with Mouse Vβ TCR Screening Panel from BD Biosciences according to manufacturer’s instructions. To stain for the intracellular activity of the translocated YopE-TEM1 fusion protein, suspended splenocytes (1X10^6^ cells) were incubated in 100 μL of complete cell medium (Dulbecco modified Eagle medium supplemented with 10% heat-inactivated fetal bovine serum, 12.5 mM HEPES, 2 mM L-glutamine, 1 mM sodium pyruvate, 1 mM penicillin-streptomycin and 55 μM β-mercaptoethanol), then 20 μL of CCF4-AM reagent (Invitrogen) in 6X Substrate Loading Solution was added to the bottom of the wells and incubated for 1 h at room temperature. The cells were then washed 5 times and incubated with appropriate antibodies. Anti-mouse antibodies used to characterize the leukocytes are PE F4/80 (BM8), PerCP/Cy5.5 Ly6C (HK1.4), PE/Cy7 CD11c (N418), Alexa Fluor 700 Ly6G (1A8), Alexa Fluor 647 I-A/I-E (M5/114.15.2), Brilliant Violet 510 CD11b (M1/70). Antibodies were from BioLegend unless indicated otherwise. Labeled cells were analyzed using a BD FACSCaliber or a Cytek DXP 8 color upgrade. Gating on side and forward scatter was used to focus on intact splenocytes. Representative examples of the gating strategy are show in S5 Fig. Data were analyzed with FlowJo software (Tree Star).

### Generation of ET-enrich CD8^+^ T cell lines and in vitro antigen presentation

ET-enriched CD8^+^ T cell lines were generated from mice that survived IV challenge with 500 to 2000 CFU of mE as described before with modification [22]. Briefly, RBC-lysed splenocytes from naïve C57BL/6 mice were treated with 50 μg/ml mitomycin C (Sigma-Aldrich) for 30 min at 37°C in complete cell medium, washed with complete medium, used at 1X10^7^ cells/well in 6-well plate as APCs. CD8^+^ T cells were enriched from spleens of mice infected with mE for 180 days using CD8a (Ly-2) MicroBeads (Miltenyi Biotec.) following manufacturer’s instructions. One million enriched CD8^+^ T cells were mixed into each well of APCs in 4 ml of complete medium containing 160 pM YopE_69-77_ peptide. After 48 hours, recombinant human IL-2 (Peprotech, Rocky Hill, NJ) was added to the culture at a final concentration of 20 U/ml. Culture media was replenished every other day with fresh IL-2-containing medium. After 2 weeks, cells were washed into fresh medium and used for in vitro antigen presentation or DEAD assay. In general, about 5–11% of total splenocytes were enriched as CD8^+^ T cells; ~2% or less of the enriched cells and 10–45% of the resulting viable ET-enriched CD8+ T cell lines stained positive for YopE_69-77_ tetramer.

For in vitro antigen presentation, BMDMs at 4X10^4^ cells/well on 96-well plate were left uninfected or infected with various *Y*. *pseudotuberculosis* strains for 4 h at MOI of 10, gentamicin was added to final concentration of 8 μg/ml after 2 h. To prepare bacteria for infection, bacteria were grown in the low Ca^2+^ condition with shaking at 28°C for 1 h and 37°C for 2 h. The bacteria were then washed and resuspended in Hank’s balanced salt solution pre-warmed to 37°C, diluted to desired CFU/ml in 100 μl BMM-low medium (Dulbecco modified Eagle medium supplemented with 10% heat inactivated fetal bovine serum, 15% L-cell conditioned medium, 2 mM L-glutamine, 1 mM sodium pyruvate), applied to the BMDMs. The plate was centrifuged for 5 min at 200X g and incubated at 37°C for 2 h, next gentamicin was added to final concentration of 8 μg/ml and the plate incubated for additional 2 h at 37°C. Control wells contained bacteria but not BMDMs, and were otherwise treated identically. ET-enriched CD8^+^ T cell lines at 1.6X10^6^/ml in 100 μl complete cell medium containing 2X penicillin-streptomycin was added to the wells containing infected BMDMs or bacteria alone. The plate was then incubated at 37°C for 48 h, and the concentrations of IFNγ in the supernatant was determined with Quantikine Mouse IFNγ kit from R&D Systems, Inc., following manufacturer’s instructions.

### Direct Ex vivo Antigen Detection (DEAD) assay

CCR2-GFP mice were used within 8 weeks to 3 months of age. Seven days after IV infection with either 1000 CFU/mouse of mE or 2X10^5^ CFU/mouse of ΔBmE, RBC were lysed and monocytes were first enriched from total splenocytes using the EasySep Mouse Monocyte Enrichment kit from Stem Cell Technologies following manufacturer’s instructions. Next, GFP+ cells were sorted from GFP- cells using BD FACSAria III. These GFP^+^ and GFP^-^ cells were plated on 96 well plates at 10^5^ cells/well, then overlaid with 4X10^5^ cloned YopE_69-77_ specific CD8^+^ T cells in complete cell medium with antibiotics. The concentrations of IFNγ in the supernatant were determined 48 h later with ELISA.

### Adoptive transfer of infDC from CCR2-GFP mice into Ccr2^-/-^ mice

Four days after IV infection of CCR2-GFP mice with 1000 CFU/mouse of ΔYopE, GFP^+^ cells were enriched and sorted as described above. The isolated GFP^+^ cells were washed three times in PBS and injected (1.8-2X10^6^ cells/mouse) retro-orbitally to Ccr2^-/-^ mice that had been infected with mE at 1000 CFU/mouse mE the day before. Control mice received PBS alone by retro-orbital injection. Seven days post-infection, splenocytes were analyzed with tetramer and antibody staining followed with flow cytometry.

### Statistical analysis

Statistical analysis was performed with Prism 5.0 (Graphpad) software, mean and SEM were plotted. The tests used are as indicated in the Fig legends. P values of less than 0.05 were considered significant.

## Supporting Information

S1 Fig Analysis of TCR Vβ subsets on CD8^+^ T cells from a control uninfected mouse. Upper panels show representative histographs of tetramer (YopE-APC) and Vβ8.1 & 8.2 (left) or Vβ8.3 (right) signals from CD8^+^ T cells from a control uninfected (UI) C57BL/6 mouse. Lower panels show data from an mE-infected mouse and are the same as shown Fig 1A. Numerical values correspond to percentages of gated cell populations among total CD8^+^ T cells.
(TIF)

S2 Fig Comparison of spleen and liver colonization by mE and ΔBmE at 4 dpi. Groups of 4 C57BL/6 mice were infected IV with 1000 CFU of mE or 5X10^5^ CFU of ΔBmE. Four dpi the colonization levels of spleen (A) and liver (B) were determined by CFU assay. Each symbol represents the value obtained from one mouse. Data shown are the results of one experiment. P values indicated were determined with Mann-Whitney test.
(TIF)

S3 Fig The YopEΔN15 mutant is defective for translocation of YopE. BMDMs were infected with mE or YopEΔN15 (ΔN15) and detergent solubility assay was performed as described in Experimental Procedures. Samples of the resulting insoluble (left, containing bacterial associated YopE) or soluble (right, containing YopE in the host cell cytosol) fractions were analyzed by immunoblotting with anti-YopE antibodies. Positions of molecular weight standards in kDa are shown on the left.
(TIF)

S4 Fig Time course analysis of ET cell numbers in C57BL/6 or Ccr2^-/-^ mice infected with mE. C57BL/6 (filled circles) or Ccr2^-/-^ (open circles) mice were left uninfected (UI) or infected IV with 1000 CFU of mE. On the indicated day, the numbers of ET cells in spleens were determined as described in Experimental Procedures. Each symbol represents the value obtained from one mouse, and the results shown are combined from 2–3 independent experiments at each time point. “***” Indicates a significant difference (P<0.0001) as compared to any other condition using one way analysis of variance followed by Bonferroni’s Multiple Comparison Test. Symbols in gray represent values that also appear in Fig 5E in the main text.
(TIF)

S5 Fig The gating strategy used in Fig 6 of main text. As described in the legend of Fig 6, groups of mice were infected with YopE-Bla or ΔB YopE-Bla for 6 days and splenocytes were analyzed with flow cytometry following CCF4-AM substrate loading and antibody staining. Representative contour plots are shown to indicate the gating of CD11b^+^ (A), CD11c^+^ (C) and CD8^+^ (Gate Q2 in E, these events are also CD3^+^) among splenocytes. Panels (B), (D) and (F) show gating used to indicate CD11b^+^, CD11c^+^ and CD8^+^ cells, respectively, that emitted blue fluorescence as a result of receiving translocated YopE-TEM1 fusion protein. (G) Representative histograph of total splenocytes indicating gating of CD11b^+^Ly6C^hi^ infDC and CD11b^+^Ly6C^med^ PMN.
(TIF)
