# Supplementary figures and images for: CCR2+ Inflammatory Dendritic Cells and Translocation of Antigen by Type III Secretion Are Required for the Exceptionally Large CD8+ T Cell Response to the Protective YopE69-77 Epitope during Yersinia Infection
Source: PLoS Pathog. 2015 Oct 15;11(10):e1005167. doi: 10.1371/journal.ppat.1005167 (PMC4607306; doi:10.1371/journal.ppat.1005167)

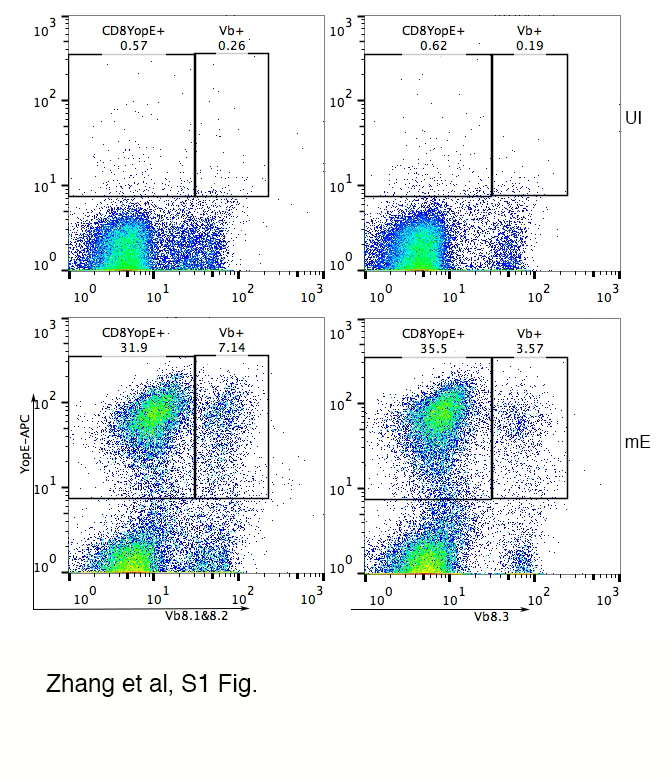

Supplement: S1 Fig — Upper panels show representative histographs of tetramer (YopE-APC) and Vβ8.1 & 8.2 (left) or Vβ8.3 (right) signals from CD8+ T cells from a control uninfected (UI) C57BL/6 mouse. Lower panels show data from an mE-infected mouse and are the same as shown Fig 1A. Numerical values correspond to percentages of gated cell populations among total CD8+ T cells. (TIF) [file ppat.1005167.s001.tif]

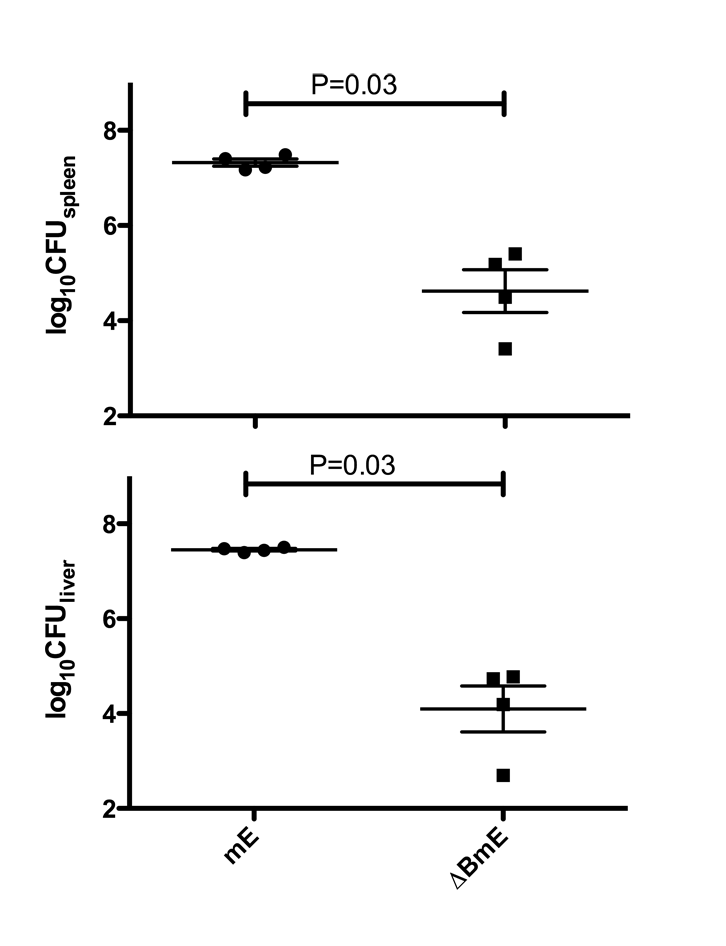

Supplement: S2 Fig — Groups of 4 C57BL/6 mice were infected IV with 1000 CFU of mE or 5X105 CFU of ΔBmE. Four dpi the colonization levels of spleen (A) and liver (B) were determined by CFU assay. Each symbol represents the value obtained from one mouse. Data shown are the results of one experiment. P values indicated were determined with Mann-Whitney test. (TIF) [file ppat.1005167.s002.tif]

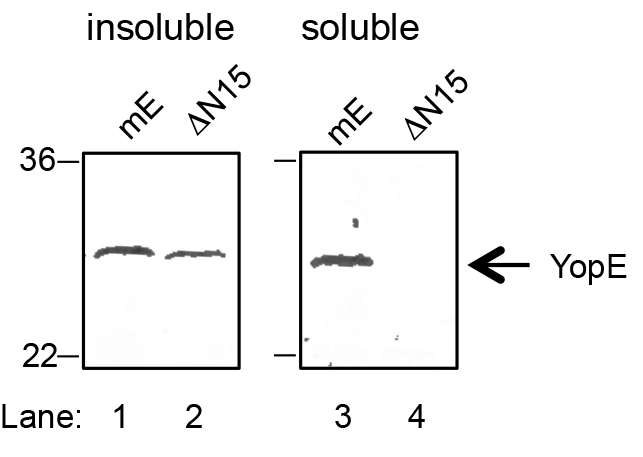

Supplement: S3 Fig — BMDMs were infected with mE or YopEΔN15 (ΔN15) and detergent solubility assay was performed as described in Experimental Procedures. Samples of the resulting insoluble (left, containing bacterial associated YopE) or soluble (right, containing YopE in the host cell cytosol) fractions were analyzed by immunoblotting with anti-YopE antibodies. Positions of molecular weight standards in kDa are shown on the left. (TIF) [file ppat.1005167.s003.tif]

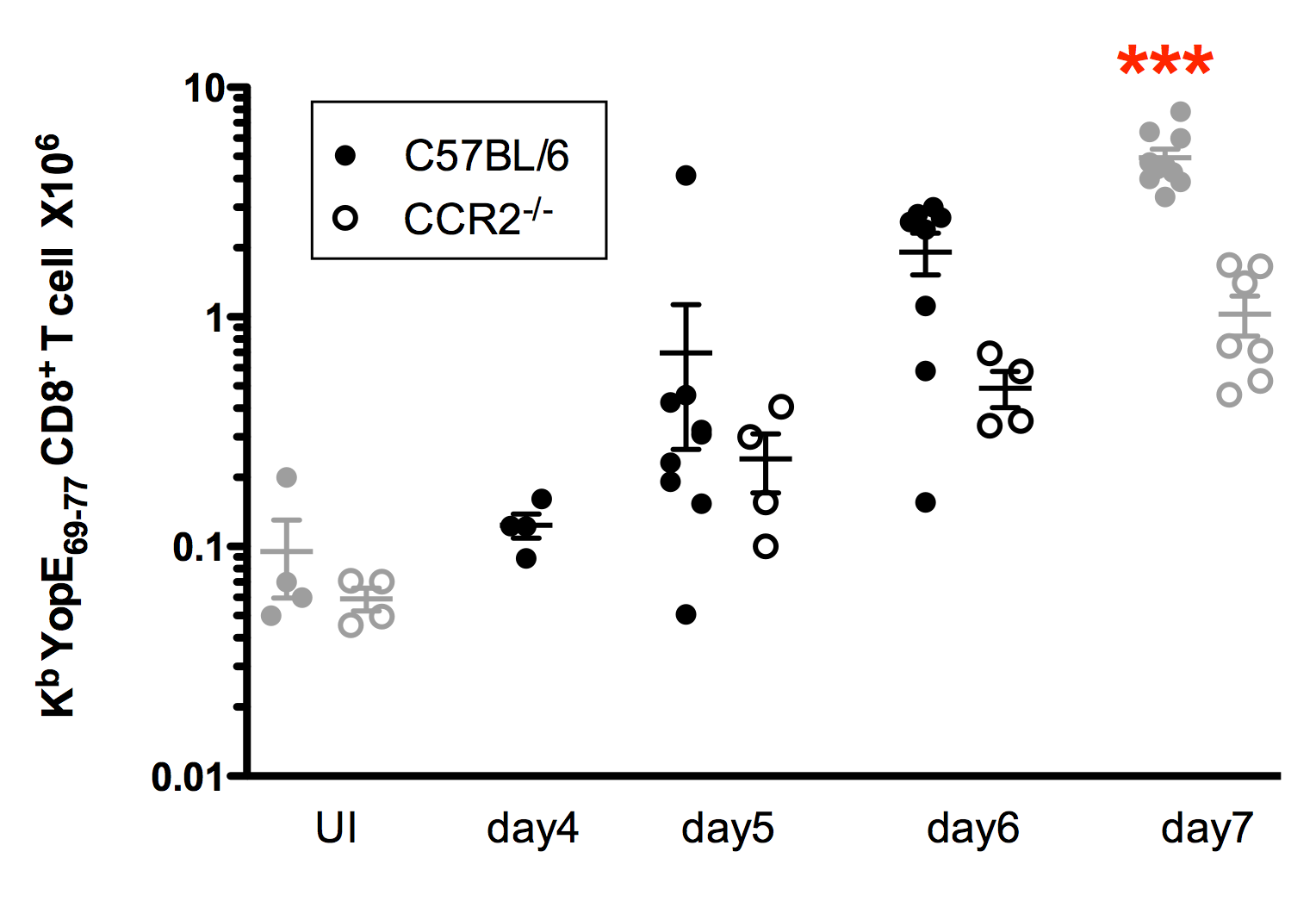

Supplement: S4 Fig — C57BL/6 (filled circles) or Ccr2-/- (open circles) mice were left uninfected (UI) or infected IV with 1000 CFU of mE. On the indicated day, the numbers of ET cells in spleens were determined as described in Experimental Procedures. Each symbol represents the value obtained from one mouse, and the results shown are combined from 2–3 independent experiments at each time point. “***” Indicates a significant difference (P<0.0001) as compared to any other condition using one way analysis of variance followed by Bonferroni’s Multiple Comparison Test. Symbols in gray represent values that also appear in Fig 5E in the main text. (TIF) [file ppat.1005167.s004.tif]

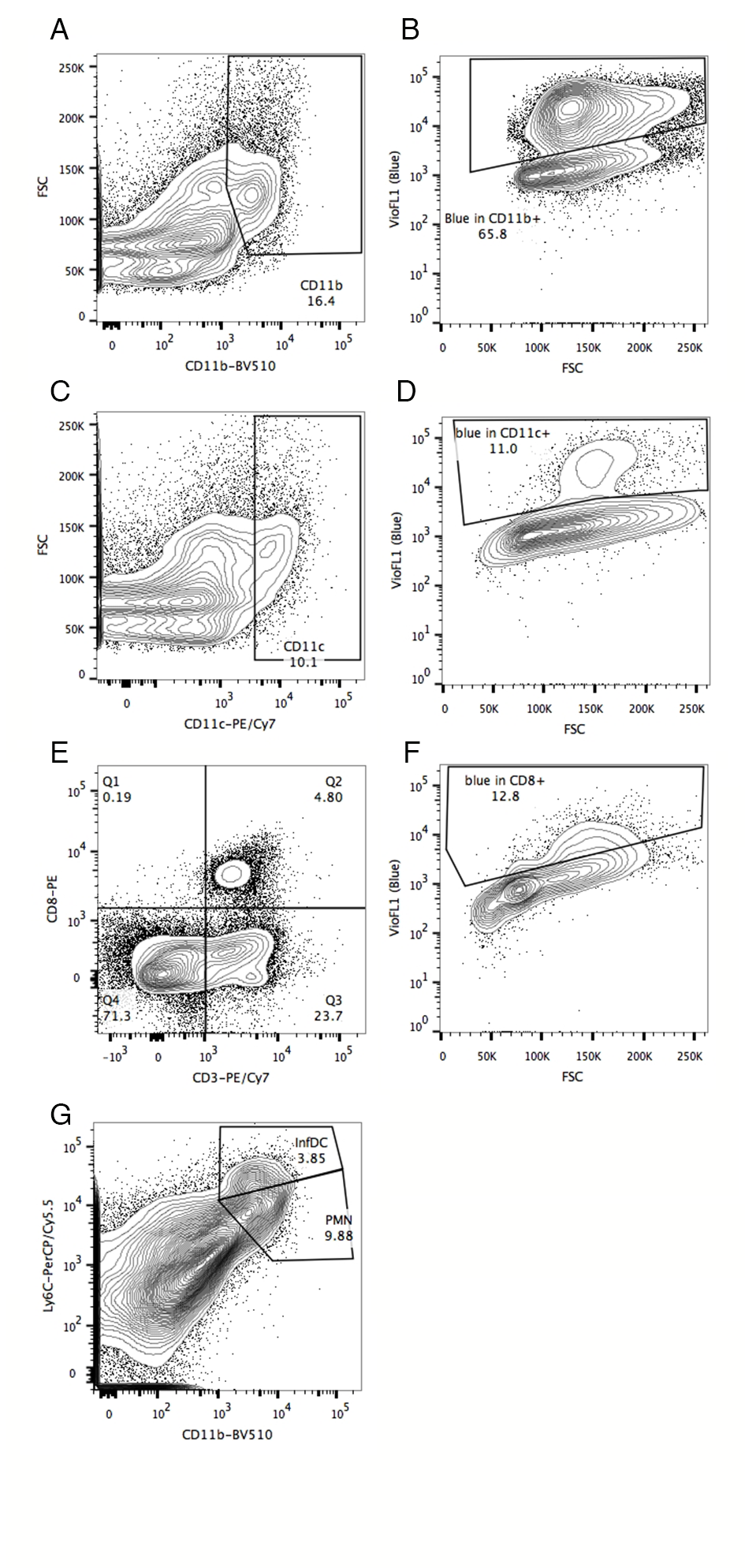

Supplement: S5 Fig — As described in the legend of Fig 6, groups of mice were infected with YopE-Bla or ΔB YopE-Bla for 6 days and splenocytes were analyzed with flow cytometry following CCF4-AM substrate loading and antibody staining. Representative contour plots are shown to indicate the gating of CD11b+ (A), CD11c+ (C) and CD8+ (Gate Q2 in E, these events are also CD3+) among splenocytes. Panels (B), (D) and (F) show gating used to indicate CD11b+, CD11c+ and CD8+ cells, respectively, that emitted blue fluorescence as a result of receiving translocated YopE-TEM1 fusion protein. (G) Representative histograph of total splenocytes indicating gating of CD11b+Ly6Chi infDC and CD11b+Ly6Cmed PMN. (TIF) [file ppat.1005167.s005.tif]
